# IFNγ-induced memory in human macrophages is sustained by the durability of cytokine signaling itself

**DOI:** 10.1084/jem.20250976

**Published:** 2026-02-18

**Authors:** Aleksandr Gorin, Siyue Niu, Noa Harriott, Vyas Koduvayur, Quen J. Cheng, Alexander Hoffmann

**Affiliations:** 1 Division of Infectious Diseases, Department of Medicine, University of California, Los Angeles, Los Angeles, CA, USA; 2 Department of Microbiology, Immunology, and Molecular Genetics, University of California, Los Angeles, Los Angeles, CA, USA; 3 Institute for Quantitative and Computational Biosciences, University of California, Los Angeles, Los Angeles, CA, USA

## Abstract

Macrophages, as key sentinel cells of the innate immune system, can retain memory of prior stimulus exposure. IFNγ plays a central role in maintaining trained immunity *in vivo* and can induce potent memory in macrophages. Such memory is associated with the formation of *de novo* enhancers that alter gene expression responses to subsequent stimuli. However, how such enhancers are maintained after cytokine exposure remains unclear. We report that the mechanism underlying durable IFNγ-induced enhancers is not cell intrinsic. IFNγ-treated macrophages continue to exhibit JAK/STAT signaling days after cytokine removal. Blocking IFNγ signaling with a JAK inhibitor or anti-IFNγ neutralizing antibodies after cytokine removal is sufficient to reverse IFNγ-induced enhancers and erase the potentiated state of the treated macrophages. Our findings suggest that epigenetic changes in macrophages do not inherently encode innate immune memory or a “potentiated” macrophage state, but in fact are themselves dependent on ongoing signaling from cytokines sequestered at the cell surface.

## Teaser

IFNγ induces long-lasting memory in macrophages, which is generally assumed to be maintained by their epigenome. Gorin et al. report that IFNγ-induced enhancers and potentiated stimulus-response capabilities are, in fact, maintained by persistent JAK/STAT signaling from cytokine sequestered at the cell surface.

## Graphical Abstract

**Figure:**
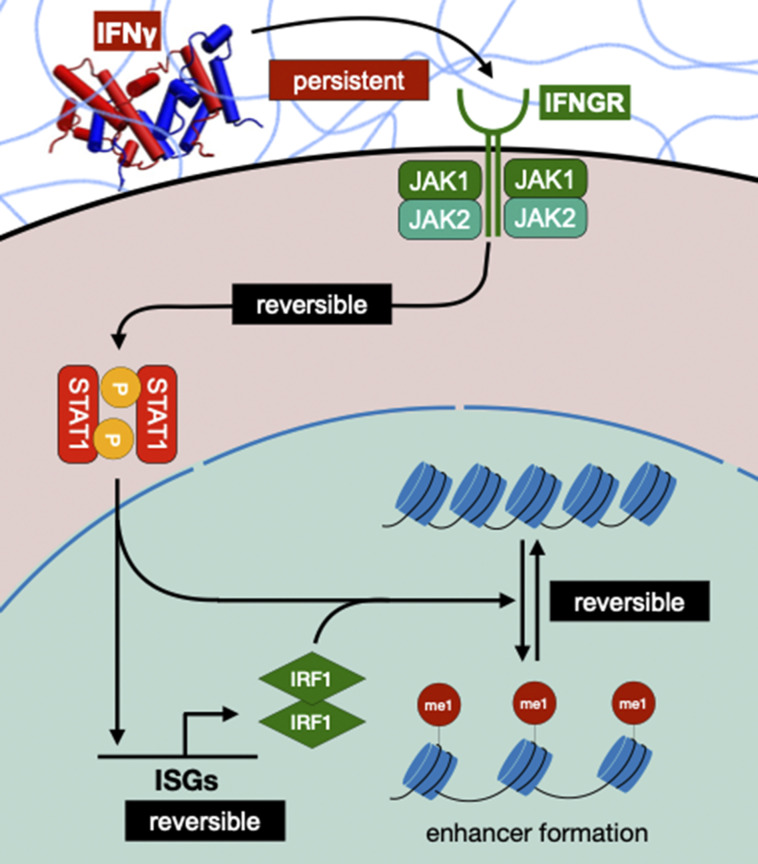

## Introduction

Innate immune memory, or the ability of the innate immune system to maintain memory of prior immune threats, is apparent in human vaccine cohorts and long-lasting immune sequelae following viral infections (Arts et al., 2018; Giamarellos-Bourboulis et al., 2020; Cheong et al., 2023; Murphy et al., 2023). Mice treated with Bacillus Calmette-Guérin (BCG), the fungal compound β-D-glucan (Kleinnijenhuis et al., 2012; Quintin et al., 2012; Ciarlo et al., 2020), or transient respiratory viral infections (Yao et al., 2018; Wang et al., 2023; Lercher et al., 2024) retain improved immunologic responses to subsequent infections even when they lack a functional adaptive immune system. Such memory can be encoded in the bone marrow, where hematopoietic progenitor cells differentiate to produce “trained” myeloid cells (Kaufmann et al., 2018). Other models have demonstrated that tissue-resident macrophages at the site of the original exposure are also capable of retaining memory and mediating improved immunologic responses on rechallenge (Yao et al., 2018; Chakraborty et al., 2023; Wang et al., 2023; Lercher et al., 2024). Recent work has repeatedly demonstrated the central importance of type II IFN, IFNγ, in such immunologic memory in macrophages (Yao et al., 2018; Li et al., 2023; Wang et al., 2023; Tran et al., 2024; Crabtree et al., 2022).

The mechanism that encodes memory in terminally differentiated macrophages remains under investigation. However, several *in vivo* studies have demonstrated altered epigenetic landscapes in trained tissue-resident macrophages. *In vitro,* macrophages are “polarized” in response to stimuli such as IFNγ; such stimulation of macrophage leads to the acquisition of durable enhancer marks (H3K4me1 and H3K4me2) that can persist long after the stimulus is removed (Kaikkonen et al., 2013; Ostuni et al., 2013; Saeed et al., 2014; Kamada et al., 2018; Cheng et al., 2021; Chavez et al., 2025). Such “*de novo*” enhancers are thought to mediate long-term memory, mediating potentiated gene expression upon restimulation of the cell (Qiao et al., 2013; Kang et al., 2019; Chavez et al., 2025). However, how these histone marks are maintained after stimulus removal remains unknown, as the modifications themselves are reversible (Hyun et al., 2017).

IFNγ signals by binding to its cognate receptor and activating JAK1/2 signaling, which in turn phosphorylates the STAT1 protein. Phosphorylated STAT1 forms homodimers (also known as γ-associated factor, GAF) which translocate to the nucleus and induce the expression of IFN-stimulated genes (ISGs), including IFN regulatory factor 1 (IRF1). We have previously demonstrated that IRF1 and GAF work in concert to remodel chromatin and lead to the formation of hundreds of *de novo* enhancers in murine and human macrophages (Chavez et al., 2025). We also showed that IFNγ-pulsed macrophages remain hyperresponsive upon restimulation with LPS days after the cytokine is removed. Here we report on the mechanism that provides durability to these IFNγ-induced epigenetic changes and the resulting capacity for potentiated gene expression responses.

## Results

### IFNγ but not LPS-induced enhancers are long-lasting in human macrophages

**Figure S1.**
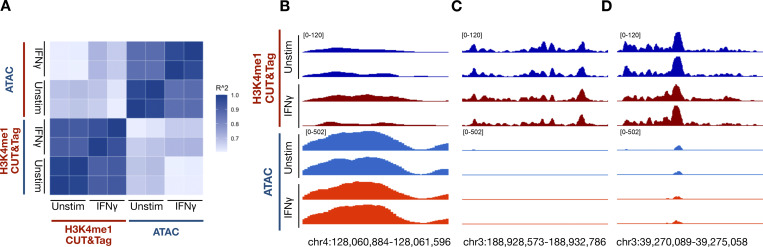
**CUT&Tag identifies H3K4me1 peaks and is distinct from ATAC.** To validate that the CUT&Tag assay for H3K4me1 identifies true histone marks over potential background Tn5 activity, we performed the ATACseq on paired samples from the same subject collected simultaneously as the CUT&Tag assay. Peaks were identified based on CUT&Tag reads, and reads within the peaks were quantified for both CUT&Tag and ATAC experiments. The results show greater consistency within each assay group rather than across, regardless of treatment. Reads are normalized across ATAC and CUT&Tag independently. **(A)** Spearman correlation of reads within the same peaks for CUT&Tag and ATAC experiments. **(B)** Genome browser track of reads within an identified CUT&Tag peak showing minimal reads within the same peak in an ATAC experiment. **(C)** Genome browser track of reads within an identified CUT&Tag peak showing higher reads within the same peak in an ATAC experiment. **(D)** Genome browser track of reads within an identified CUT&Tag peak showing similar reads within a peak between ATAC and CUT&Tag experiments.

**Figure 1.**
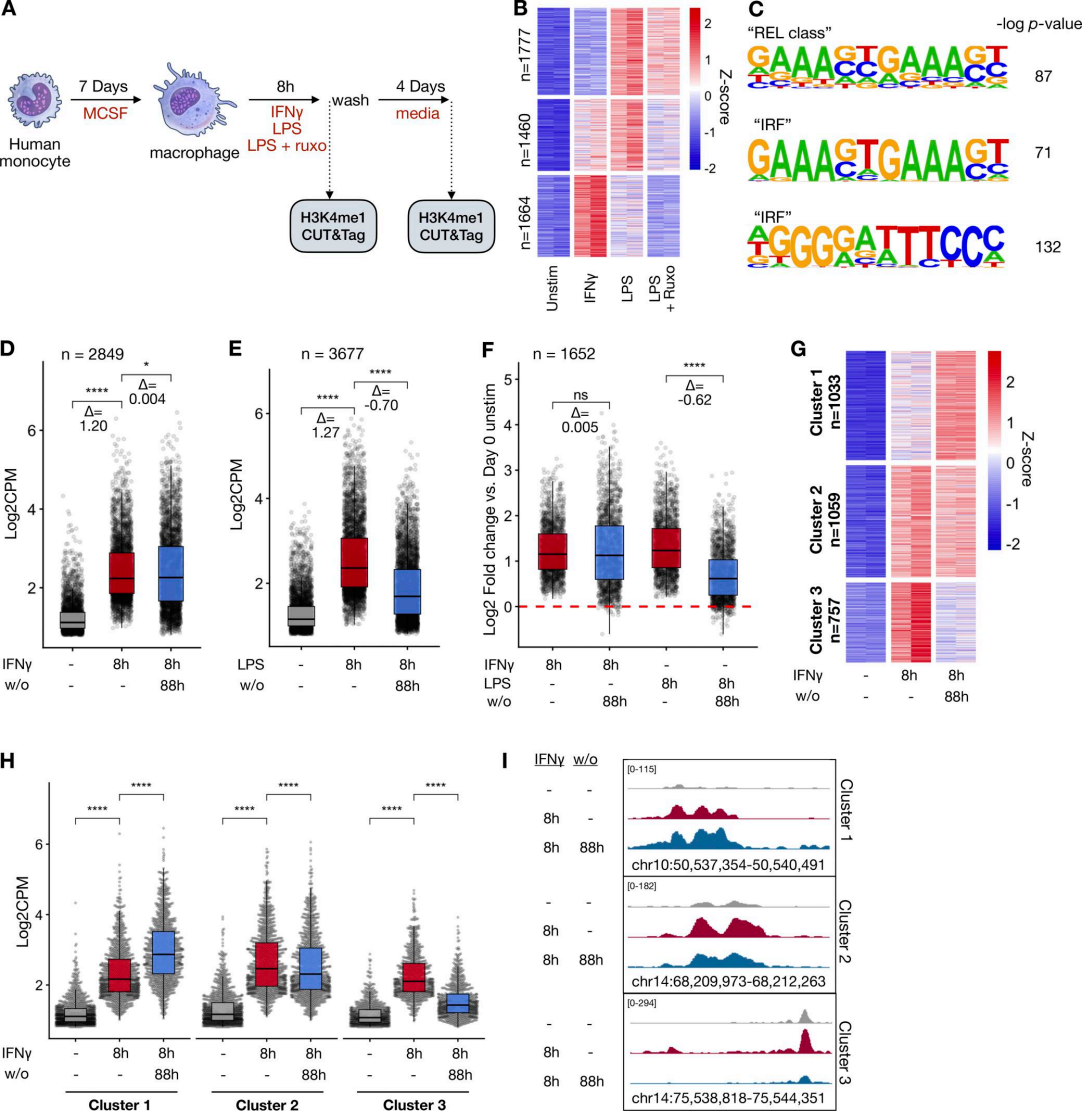
**LPS and IFNγ both generate stimulus-specific *de novo* enhancers in human macrophages; however, only IFNγ-induced enhancers are durable. (A)** Schematic of experimental design: Human macrophages were stimulated with either IFNγ (100 ng/ml), LPS (100 ng/ml), or LPS in the presence of 1 µM ruxolitinib for 8 h. Cells were subsequently washed and cultured for an additional 88 h. H3K4me1 CUT&Tag was performed at each time point. **(B)** Heatmap of Z-scored reads within H3K4me1 peaks induced by either LPS or IFNγ (L2FC > 2, FDR < 0.01). Clusters were generated by unsupervised k-means clustering. Each column represents a biological replicate from the same human donor. **(C)** Top enriched motifs in clusters from B. **(D)** Box/whisker plot quantifying log2 cpm of reads within IFNγ-induced peaks before and after cytokine washout. **(E)** Box/whisker quantifying log2 cpm of reads within LPS-induced peaks before and after cytokine washout. **(F)** Box/whisker quantifying log2 cpm of reads within peaks induced by both IFNγ and LPS (L2FC > 2, FDR < 0.01 for each) peaks before and after cytokine washout. **(G)** Z-scored heatmap of reads within CUT&Tag peaks of only IFNγ-induced peaks before and after cytokine washout. **(H)** Boxplot of log2 cpm of reads within peaks for each cluster identified in G. **(I)** Examples of genome browser tracks for each cluster in E. Box/whisker plots indicate interquartile range and 1.5× interquartile range. Statistical tests were determined by paired Wilcoxon test. *P < 0.05; ****P < 0.0001.

**Figure S2.**
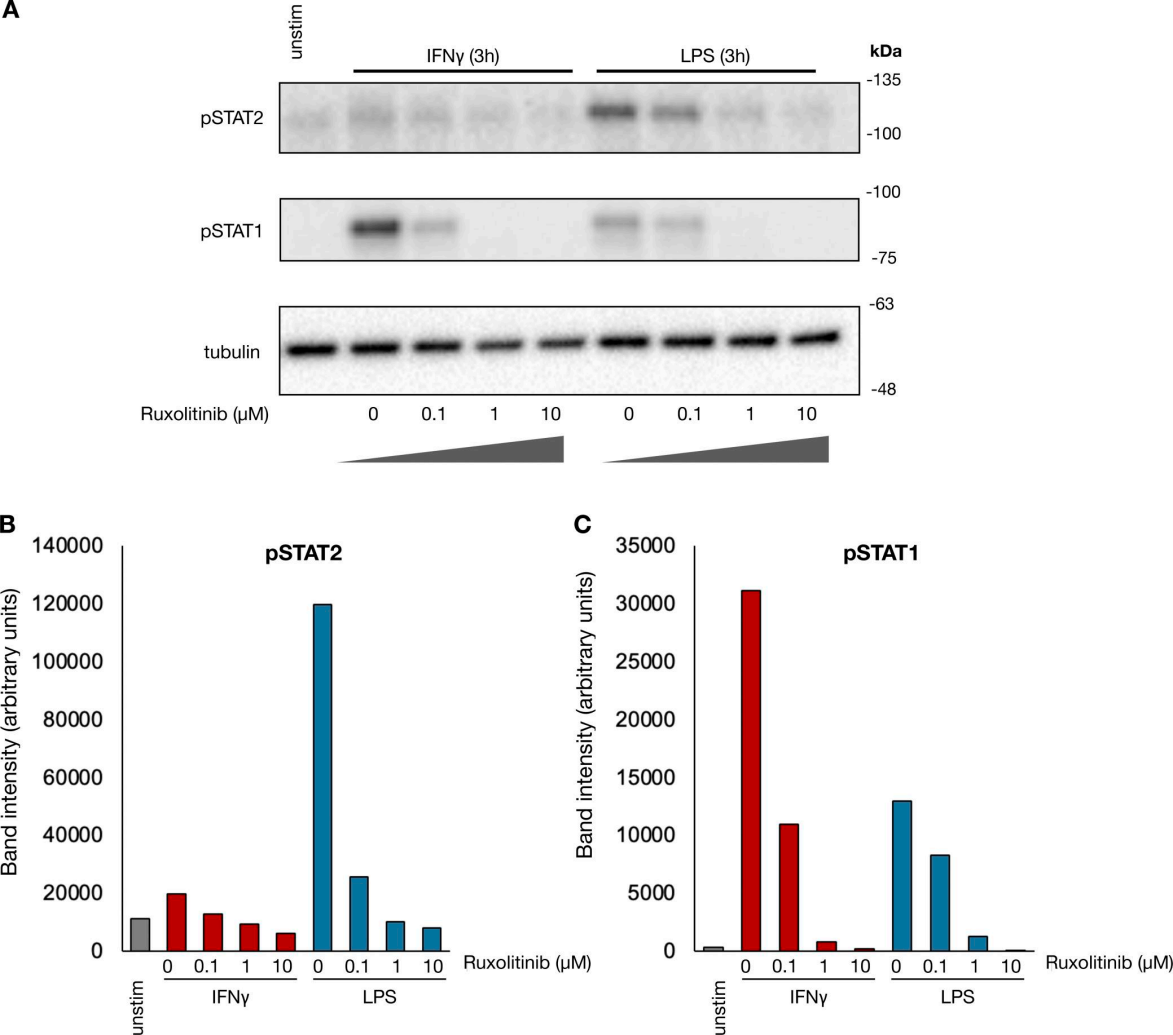
**Ruxolitinib blocks LPS and IFNγ-induced Janus kinase signaling. (A)** Human macrophages were pre-treated with increasing concentrations of ruxolitinib for 15 min and subsequently stimulated with IFNγ (100 ng/ml) or LPS (100 ng/ml) for 3 h. Whole-cell lysate western blots showing effect of ruxolitinib on STAT1 and STAT2 phosphorylation by each stimulus. Blot is representative of two replicates from separate human donors. **(B and C)** Quantification of pSTAT2 and (C) pSTAT1 band intensities in A. Source data are available for this figure: SourceData FS2.

We first asked whether human monocyte–derived macrophages gain enhancer marks in a stimulus-specific manner. After validating the reliability of the cleavage under targets and tagmentation (CUT&Tag) assay (Fig. S1), we stimulated human macrophages with IFNγ or LPS for 8 h and performed H3K4me1 CUT&Tag. LPS activates cells via direct TLR signaling as well as the IFNβ–JAK/STAT signaling axis. To identify JAK/STAT-dependent LPS enhancers, we treated macrophages (Fig. 1 A) with LPS in the presence of the JAK inhibitor ruxolitinib at a dose (1 µM) sufficient to block IFNβ–JAK/STAT signaling (Fig. S2). We were able to identify 2849 IFNγ and 3677 LPS-induced *de novo* enhancers (induced H3K4me1 peaks). Unsupervised k-means clustering separated the *de novo* enhancers into three major groups (Fig. 1 B and Data S1): an LPS-specific/JAK-independent cluster, a cluster of JAK-dependent enhancers shared by LPS and IFNγ, and an IFNγ-specific cluster. Motif analysis of each cluster showed that the most-enriched motif in the LPS-specific/JAK-independent cluster was the NFκB “REL” class, while the “IRF1” motif was most highly enriched for the JAK-dependent LPS cluster and IFNγ-specific cluster (Fig. 1 C).

Next, we explored enhancer durability after stimulus withdrawal. Macrophages were washed after IFNγ or LPS stimulation, and the cells were cultured for an additional 88 h (4 days) in fresh media before performing H3K4me1 CUT&Tag. IFNγ-induced *de novo* enhancers showed persistence after washout (Fig. 1 D); in contrast, we observed that LPS-induced *de novo* enhancers showed a significant decrease back toward baseline after the stimulus was removed (Fig. 1 E). We asked whether *de novo* enhancers shared between the two stimuli behave differently after washout; limiting our analysis to the 1652 *de novo* enhancers that were induced by both LPS and IFNγ confirmed that IFNγ-induced enhancers persisted (mean Log2 fold change [L2FC] = 0.005, P = 0.31) after washout, while LPS-induced enhancers reverted toward baseline (mean L2FC = −0.63, *p* < −2^−16^) (Fig. 1 F). Further, unsupervised k-means clustering of the IFNγ-induced enhancers demonstrated three major patterns of behavior after cytokine washout (Fig. 1, G and I; and Data S2): one cluster where enhancer marks persisted after washout, one where they decreased after washout, and a third that showed a further increase in the H3K4me1 signal.

### IFNγ induces long-lasting chromatin accessibility and IRF1/STAT1 activity

**Figure 2.**
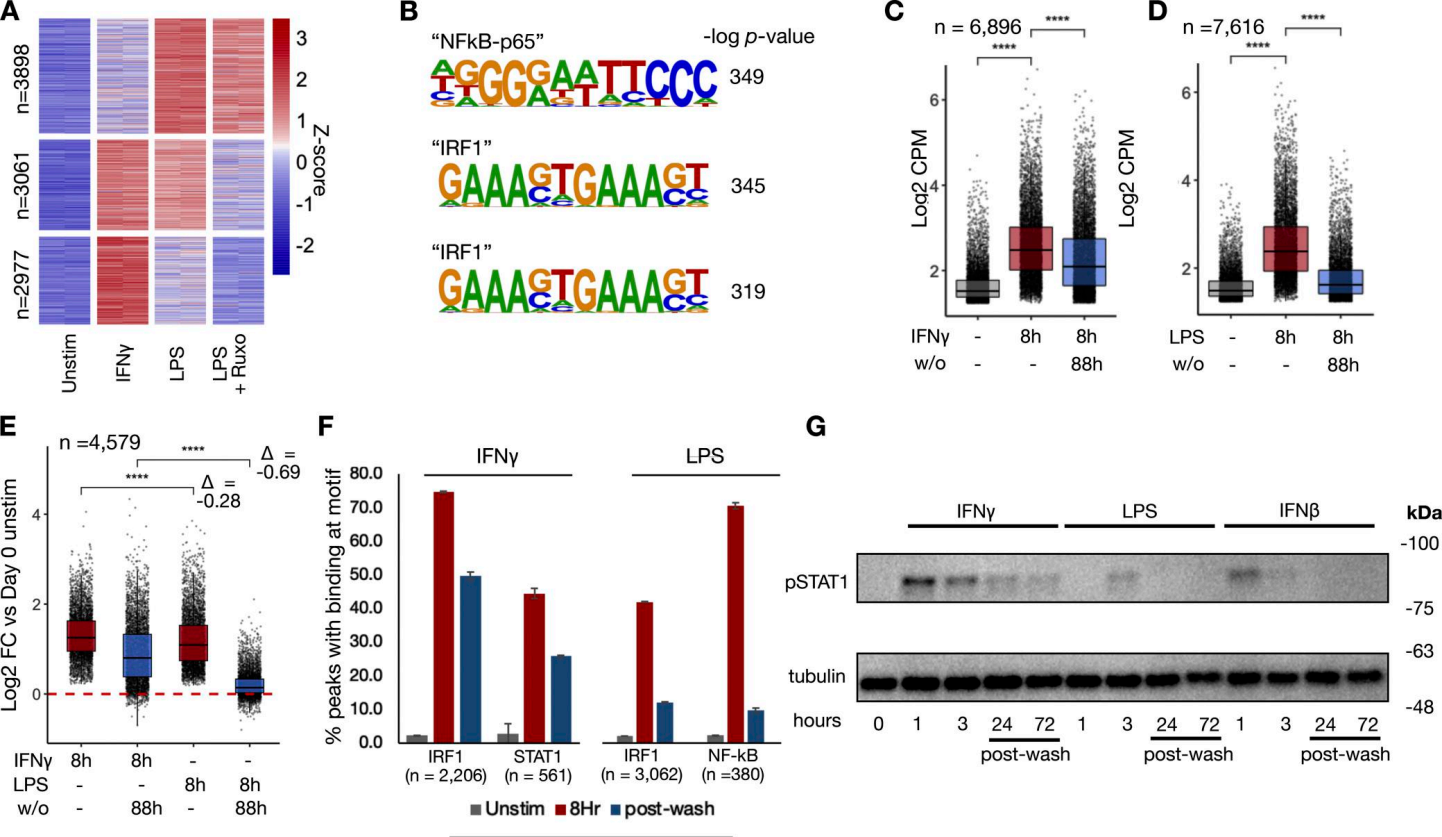
**IFNγ induces long-lasting transcription factor activity and chromatin accessibility after washout.** Macrophages were treated with LPS, IFNγ, and LPS in the presence of ruxolitinib for 8 h, as in Fig. 1 A. Cells were washed and cultured for an additional 88 h. ATACseq was performed after 8 h of stimulation and 4 days after washout. **(A)** Heatmap of Z-scored reads within ATAC peaks induced by either LPS or IFNγ (L2FC > 2, FDR < 0.01). Clusters were generated by unsupervised k-means clustering. Each column represents a biological replicate from the same human donor. **(B)** Top enriched motifs in clusters from A. **(C)** Boxplot quantifying log2 cpm of reads within IFNγ-induced ATAC peaks before and after cytokine washout. **(D)** Boxplot quantifying log2 cpm of reads within LPS-induced ATAC peaks before and after cytokine washout. **(E)** Boxplot quantifying log2 cpm of reads within ATAC peaks induced by both IFNγ and LPS (L2FC > 2, FDR < 0.01 for each) peaks before and after cytokine washout. **(F)** Barplot quantifying percent of transcription factor-bound motifs within STAT1 and IRF1 (IFNγ) and IRF1 and NF-κB (LPS) within induced ATAC peaks in C and D for unstimulated, IFNγ/LPS-stimulated macrophages, and stimulated macrophages 4 days after washout. Motif binding predicted using TOBIAS ATACseq footprinting analysis. Results are average of two technical replicates from a single subject; error bars display standard deviation. **(G)** Human macrophages were stimulated with IFNγ (100 ng/ml), LPS (100 ng/ml), or IFNβ (10 ng/ml) for 8 h, washed, and then cultured for an additional 66 h. Cells were collected, and whole cell western blotting for phosphorylated STAT1 was performed at the indicated time points. Blot is representative of three replicates from two separate human donors. All box/whisker plots indicate interquartile range and 1.5× interquartile range. Statistical tests were determined by paired Wilcoxon test. ****P < 0.0001. Source data are available for this figure: SourceData F2.

To determine whether chromatin opening precedes enhancer formation, as it does in murine bone marrow-derived macrophages (BMDMs) (Ostuni et al., 2013; Cheng et al., 2021; Chavez et al., 2025), we performed assay for transposase-accessible chromatin sequencing (ATACseq) on human macrophages stimulated with IFNγ, LPS, or LPS in the presence of ruxolitinib. The results revealed stimulus-specific patterns of chromatin opening, with 7,616 peaks induced by LPS and 6,896 by IFNγ (Fig. 2 A). Unsupervised k-means clustering identified LPS-specific/JAK-independent peaks, peaks shared by LPS and IFNγ that are JAK-dependent, and IFNγ-specific peaks. Motif analysis showed the “NF-κB p65” motif as most enriched in LPS-specific/JAK independent clusters, while IRF1 was most enriched in IFNγ-specific clusters and JAK-dependent LPS cluster (Fig. 2 B and Data S3).

To examine whether the chromatin accessibility was similarly transient as reported for LPS-stimulated BMDMs, cells were washed after 8 h of stimulation and cultured for an additional 88 h, when they were again collected for ATACseq. Both IFNγ (Fig. 2 C) and LPS-induced (Fig. 2 D) peaks showed a decrease in ATAC accessibility after stimulus washout; however, the decrease was less pronounced with IFNγ. Indeed, peaks shared between both stimuli demonstrated more persistence after IFNγ washout (Fig. 2 E), whereas after LPS washout, most peaks largely had reverted to baseline (L2FC = −0.28 for IFNγ vs. −0.69 for LPS).

Next, we asked whether persistent transcription factor activity after washout may be mediating persistent chromatin accessibility after IFNγ washout. Transcription factor footprinting analysis by TOBIAS (Bentsen et al., 2020) demonstrated STAT1 and IRF1 binding at accessible chromatin, with the majority of induced binding persisting 4 days after IFNγ washout (66% and 58%, respectively). In contrast, LPS-induced IRF1 and NFκB binding were decreased to only 29% and 14% of their maximum levels 4 days after washout (Fig. 2 F).

**Figure S3.**
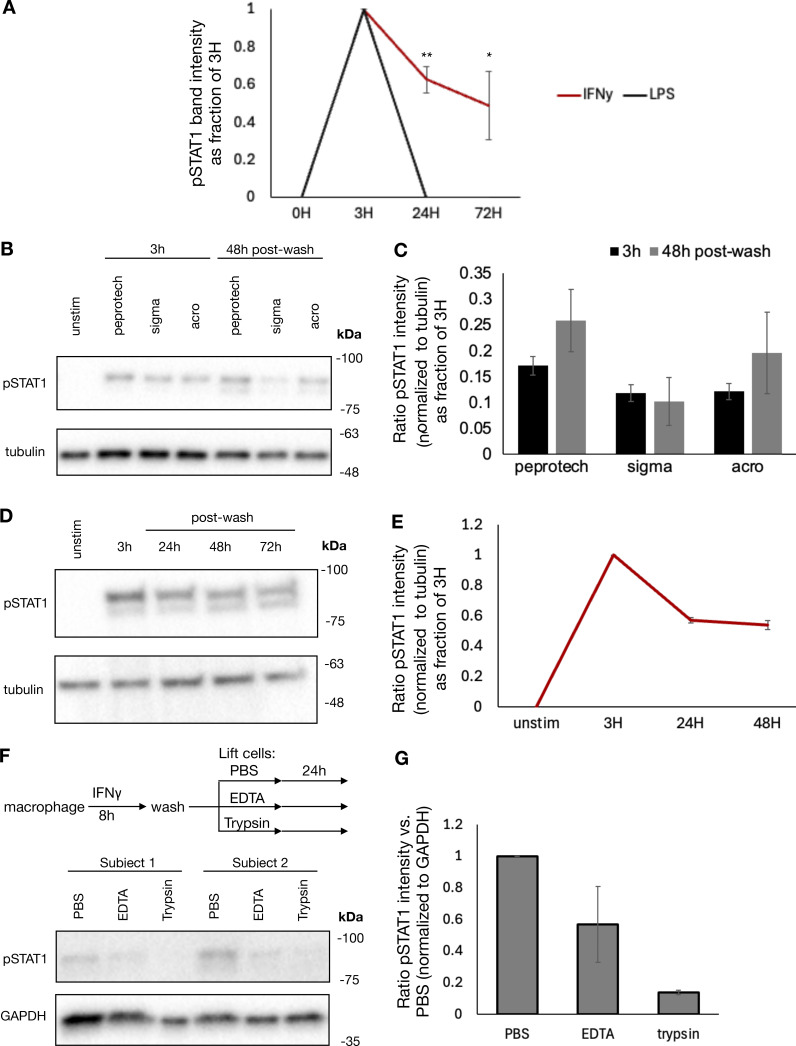
**Cell surface–bound IFNγ mediates persistent signaling regardless of cytokine manufacturer and can be degraded by trypsinization. (A)** Quantification of pSTAT1 intensity normalized to 3H IFNγ in Fig. 2 G. **(B)** Human macrophages were treated with *Escherichia coli* sourced IFNγ purchased from PeproTech and mammalian-sourced IFNγ purchased from Sigma-Aldrich and ACRO. Cells were treated at 100 ng/ml for 8 h, washed, and cultured for an additional 48 h in regular media prior to collection. Cells were collected for immunoblot at the indicated times. Representative blot of duplicates from one human subject. **(C)** Quantification of pSTAT1 band intensity normalized to tubulin at each time point in B. **(D)** Human A549 airway epithelial cells were stimulated with 100 ng/ml IFNγ, washed, and cultured for an additional 72 h. Cells were collected for immunoblot at the indicated timepoints. Representative blot of two replicates. **(E)** Quantification of pSTAT1 band intensity normalized to tubulin at each time point in D. **(F)** Human macrophages were treated with 100 ng/ml IFNγ for 8 h, washed, and lifted by scraping after incubation with either PBS, 0.5 mM EDTA in PBS, or trypsin. Cells were replated and cultured for an additional 24 h in regular media. Cells were collected for immunoblot at the indicated times. **(G)** Quantification of pSTAT1 band intensity normalized to GAPDH for each condition in F. Statistical tests were determined by single-tailed *t* test. *P < 0.05; **P < 0.01. Source data are available for this figure: SourceData FS3.

Given the durability of chromatin accessibility induced by IFNγ, we examined whether IFNγ-induced signaling in the form of phosphorylated STAT1 or IRF1 expression might persist after cytokine washout. We stimulated macrophages with IFNγ, LPS, and IFNβ for 8 h, washed out the stimulus, and cultured the cells for 3 days. Immunoblotting showed that acute treatment with each stimulus could induce STAT1 phosphorylation. This phosphorylation did not persist after washout of IFNβ and LPS; however, IFNγ-induced STAT1 phosphorylation persisted for 3 days after the washout (Fig. 2 G and Fig. S3 A).

### IFNγ signaling persists after cytokine washout due to cell capture

**Figure 3.**
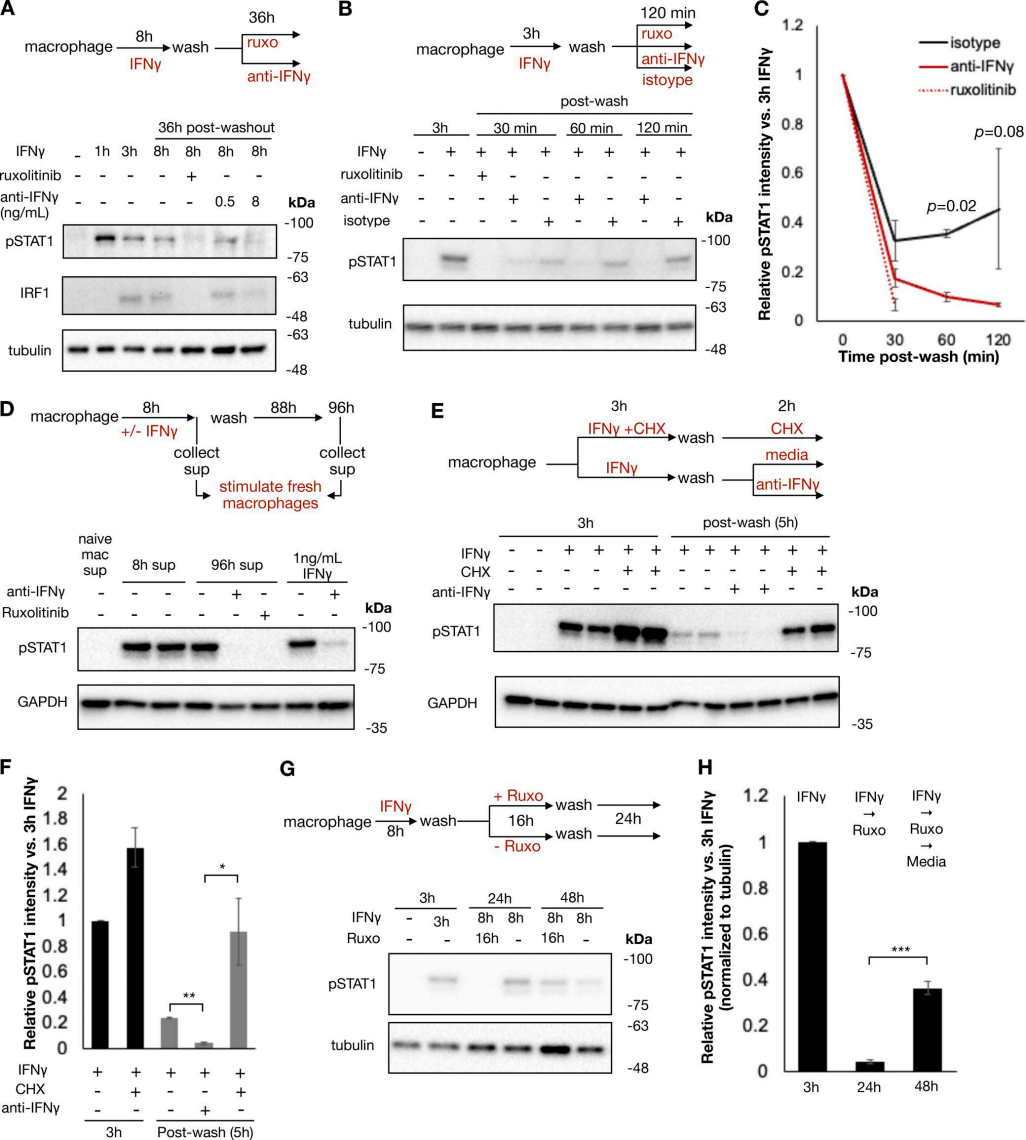
**Cell surface–bound IFNγ mediates persistent JAK/STAT signaling even after cytokine washout. (A)** Human macrophages were stimulated with IFNγ (100 ng/ml) for 8 h, washed, and then cultured in regular media or media containing ruxolitinib (1 µM) or increasing concentrations of anti-IFNγ neutralizing antibody for an additional 28 h. Cells were collected, and whole cell western blotting for phosphorylated STAT1 and IRF1 was performed at indicated time points. Representative blot of duplicates from two separate subjects. **(B)** Human macrophages were stimulated with 100 ng/ml IFNγ for 3 h, washed, and then cultured in either ruxolitinib (1 µM), anti-IFNγ neutralizing antibody (10 µg/ml), or isotype control antibody (10 µg/ml) for 2 h. Samples were collected at the indicated times for immunoblot. Representative blot of duplicates from two separate subjects. **(C)** Quantification of pSTAT1 band intensities from B. **(D)** Human macrophages were stimulated with 100 ng/ml IFNγ for 8 h, washed, and cultured for an additional 88 h in regular media. Supernatants from stimulated macrophages were collected after the 8-h stimulation and 88 h after washout. This supernatant was used to stimulate fresh macrophages for 1 h in the presence/absence of ruxolitinib (1 µM) or anti-IFNγ neutralizing antibody (10 µg/ml). As a control, fresh macrophages were stimulated with media supplemented with 1 ng/ml IFNγ for 1 h. Representative blot of duplicates from two separate subjects. **(E)** Macrophages were left in regular media or pre-treated with 10 µg/ml CHX for 15 min and stimulated with 100 ng/ml IFNγ for 3 h. Treated macrophages were washed and subsequently cultured for 2 h in regular media, media supplemented with 10 µg/ml CHX, or anti-IFNγ neutralizing antibody (10 µg/ml) and collected for immunoblot. Duplicates from one subject are shown. **(F)** Quantification of pSTAT1 band intensities in E normalized to band intensity of macrophages treated with IFNγ for 3 h. **(G)** Human macrophages were stimulated with 100 ng/ml IFNγ for 8 h, washed, and cultured in regular media or media supplemented with 1 µM ruxolitinib for 16 h. After 16 h, cells were washed again and cultured in regular media for an additional 24 h. Cells were collected for immunoblot at indicated times. Representative blot of four replicates from two subjects. **(H)** Quantification of pSTAT1 band intensities in G normalized to band intensity of macrophages treated with IFNγ for 3 h. Statistical tests were determined by a single-tailed *t* test. *P < 0.05, **P < 0.01, and ***P < 0.001. Source data are available for this figure: SourceData F3.

Previous studies have shown that IFNγ has inherent affinity for extracellular proteoglycans and phosphatidylserine present on the cell surface of cells from where it may be slowly released to mediate persistent signaling (Oyler-Yaniv et al., 2017). To evaluate whether ongoing IFNγ signaling at the cell surface was sufficient to explain the persistent STAT1 phosphorylation in our experiment, we treated macrophages with IFNγ for 8 h, followed by a wash and then culture in the presence or absence of ruxolitinib or anti-IFNγ neutralizing antibody for 36 h. Persistent STAT1 phosphorylation and IRF1 expression were observed after washout when cells were cultured in media alone; however, both were abrogated by ruxolitinib or high-dose anti-IFNγ antibody (Fig. 3 A). We observed that both ruxolitinib and anti-IFNγ antibodies lead to a rapid loss of pSTAT1 after washout, within 30 and 60 min, respectively. This effect was not dependent on macrophage Fc receptor signaling, as treatment with control isotype antibody after washout allowed for persistent pSTAT1 levels (Fig. 3, B and C). To ensure that persistent pSTAT1 postcytokine washout was not dependent on our preparation of IFNγ, we repeated this experiment with three different preparations of human IFNγ (one sourced from bacterial cells and two from mammalian cell lines), all of which showed persistent pSTAT1 48 h after cytokine washout (Fig. S3, B and C). We also tested whether our findings apply to cell types other than macrophages. Human A549 airway epithelial cells showed persistent pSTAT1 levels for at least 3 days after washout (Fig. S3, D and E).

To confirm that the persistent pSTAT1 levels were mediated by IFNγ and not secondary cytokines stimulated in response to IFNγ, we performed several experiments. First, we stimulated macrophages with IFNγ as before, washed the cells thoroughly, and then cultured for an additional 4 days. We collected supernatant from these cells either immediately prior to washout (containing 100 ng/ml of IFNγ) or at the completion of 96 h in culture. We applied these supernatants to fresh macrophages and probed for pSTAT1. Both supernatants rapidly induced pSTAT1 in fresh macrophages, and this activation was readily blocked by both ruxolitinib and anti-IFNγ antibody with the 96-h supernatant. These results confirmed that IFNγ was the only cytokine present in the supernatant after washout that could lead to STAT1 phosphorylation (Fig. 3 D). To further validate that secondary cytokines were not involved in the maintenance of pSTAT1, we treated macrophages with cycloheximide (CHX) to block *de novo* protein synthesis prior to IFNγ stimulation. CHX-treated cells and untreated cells were stimulated with IFNγ, washed, and then cultured in regular media or media supplemented with CHX or anti-IFNγ neutralizing antibody for an additional 3 h. We observed that while the neutralizing antibody readily abrogated pSTAT1 persistence after washout, CHX did not (Fig. 3, E and F). Indeed, CHX actually lead to increased pSTAT1 induction and persistence, presumably due to a reduction of negative feedback control by, for example, SOCS proteins. We also asked whether pSTAT1 signaling after washout could be blocked by terminating extracellular molecular mechanisms: we observed that trypsin digestion of IFNγ-treated macrophages dramatically lowered pSTAT1 levels, compared with macrophages lifted with PBS or EDTA (Fig. S3, F and G).

Having observed that IFNγ could not be washed off cells and mediated persistent signaling, we reasoned that STAT1 phosphorylation in IFNγ- and ruxolitinib-treated cells would resume if the JAK inhibitor was washed off. To test this, we treated macrophages with IFNγ, washed the cells, and then cultured them with ruxolitinib for 24 h. After 24 h, these cells were then washed again to remove ruxolitinib and cultured for an additional 24 h. As expected, ruxolitinib treatment blocked STAT1 phosphorylation, yet pSTAT1 returned 24 h after the ruxolitinib was washed out (Fig. 3, G and H). This result suggests that signaling persists at the level upstream of JAK and thus points to the cytokine–receptor interactions *per se*.

### The durability of IFNγ-induced chromatin opening and ISG expression relies on persistent JAK/STAT signaling

**Figure 4.**
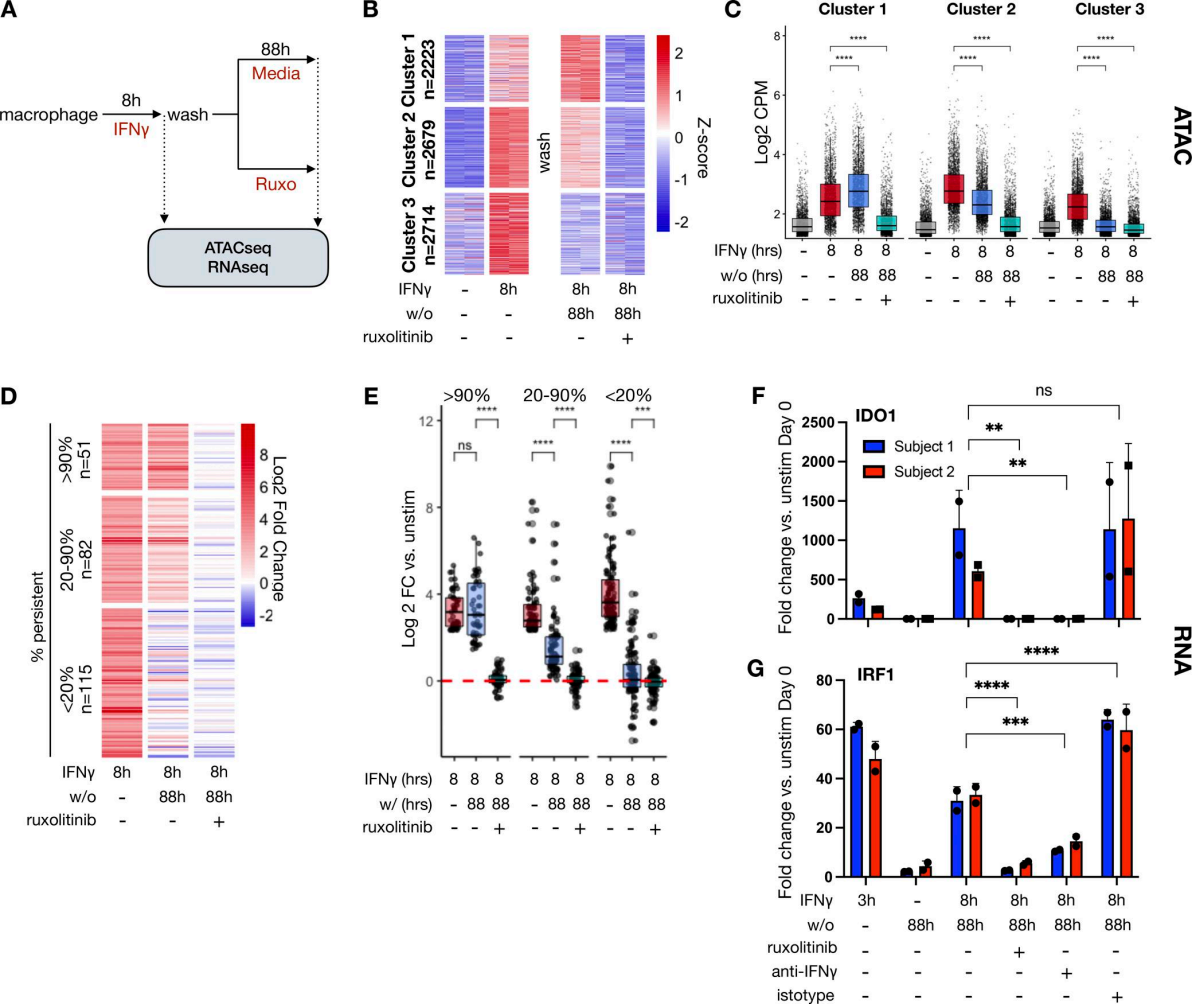
**Extracellular IFNγ signaling sustains chromatin accessibility and ISG expression even after cytokine washout. (A)** Schematic of experiments: Human macrophages were stimulated with 100 ng/ml IFNγ for 8 h, washed, and then cultured for an additional 88 h in regular media or media with 1 µM ruxolitinib for an additional 88 h. Cells were collected for ATACseq and RNAseq at the indicated time points. **(B)** Heatmap of Z-scored reads within ATAC peaks induced by IFNγ (L2FC > 2, FDR < 0.01) after 8 h of stimulation for 4 days after washout when cultured in regular media or media with 1 µM ruxolitinib. Clusters were generated by unsupervised k-means clustering. Each column represents a biological replicate from the same human donor. **(C)** Boxplot of log2CPM of reads within each peak for each cluster in B. **(D)** Heatmap of Log2 fold change in RNAseq reads of genes induced at least fivefold after 8 h of IFNγ stimulation. Log2 fold changes are shown after washout for cells cultured in regular media and media containing 1 µM ruxolitinib. Genes are clustered by persistent level of expression after washout (CPM after wash as percent of CPM at 8-h simulation). **(E)** Boxplot showing Log2 fold changes of individual genes by cluster in D. Box/whisker plots indicate interquartile range and 1.5× interquartile range. Statistical tests were determined by paired Wilcoxon test. **(F)** Macrophages were stimulated and washed as above in A; after washout, cells were cultured in media alone, media with 1 µM ruxolitinib, or 10 µg/ml anti-IFNγ neutralizing antibody for 88 h. Cells were collected 88 h after washout, and qPCR was performed for IDO1. Boxplots indicate 2^ΔΔCt^ normalized to HPRT. Error bars indicate standard deviation. Statistical tests determined by ordinary one way ANOVA. **(G)** qPCR for IRF1 as in F. ** P < 0.01; ***P < 0.001; ****P < 0.0001.

**Figure S4.**
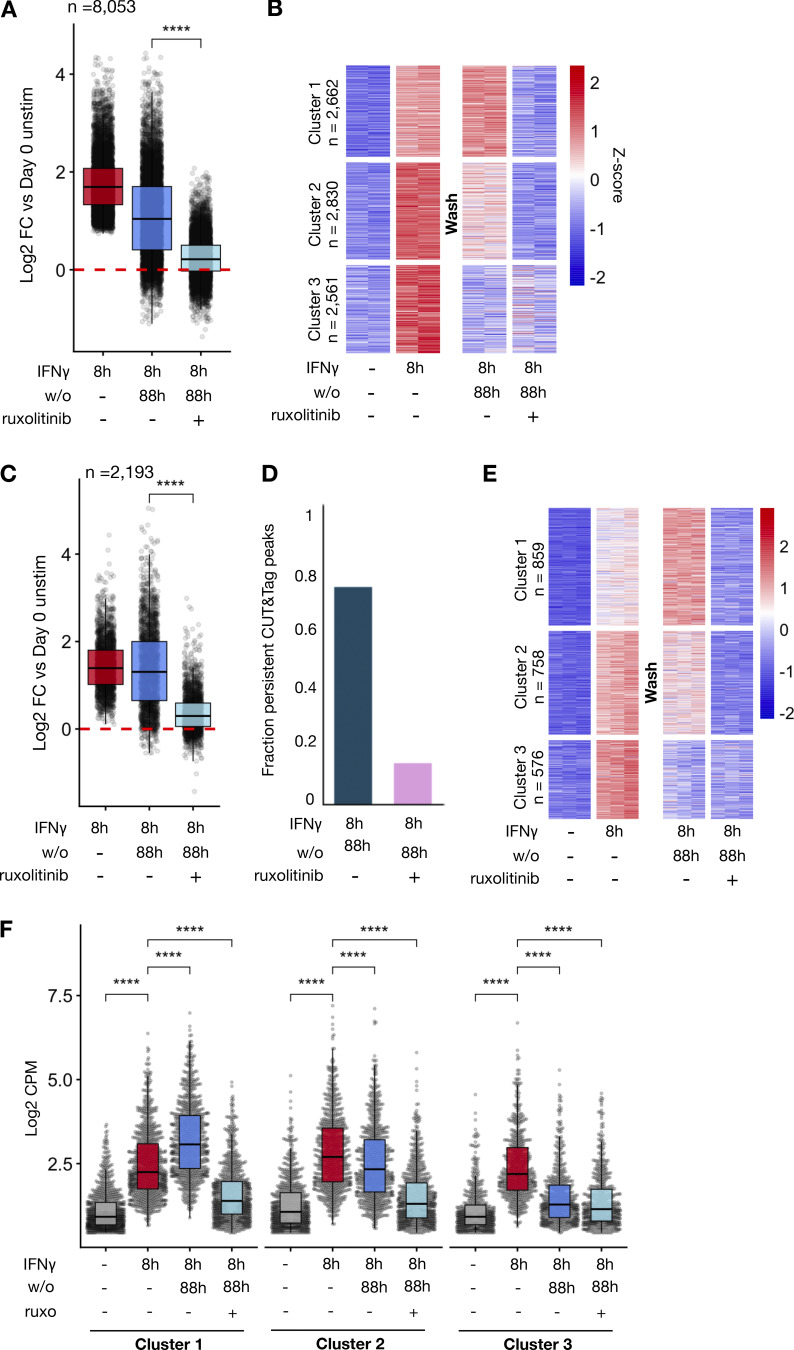
**Macrophages from a second human donor show reversibility of IFNγ-induced chromatin accessibility and *de novo* enhancers.** Human macrophages from a second human subject were stimulated with IFNγ (100 ng/ml) for 8 h. Cells were subsequently washed and cultured for an additional 88 h in standard media or media supplemented with 1 µM ruxolitinib. ATAC and H3K4me1 CUT&Tag was performed after 8 h of stimulation and 88 h after cytokine washout. **(A)** Boxplot quantifying log2 fold changes of reads within IFNγ-induced ATAC peaks after 8 h of IFNγ stimulation and after washout for each condition (L2FC > 2, FDR < 0.01). **(B)** Heatmap of Z-scored reads within ATAC peaks induced IFNγ after 8 h of stimulation and 4 days after washout for each condition. Clusters were generated by unsupervised k-means clustering. Each column represents a biological replicate from the same human donor. **(C)** Boxplot quantifying log2 fold changes of reads within IFNγ-induced H3K4me1 CUT&Tag peaks after 8 h of IFNγ stimulation and after washout for each condition (L2FC > 2, FDR < 0.01). **(D)** Barplot showing fraction of IFNγ-induced H3K4me1 peaks at 8 h that persist 4 days after washout in each condition. Persistence was defined as L2FC ≥ 0, FDR < 0.01. **(E)** Heatmap of Z-scored reads within H3K4me1 peaks induced IFNγ after 8 h of stimulation and 4 days after washout for each condition. Clusters were generated by unsupervised k-means clustering. Each column represents a biological replicate from the same human donor. **(F)** Boxplot of log2CPM of reads within each peak for each cluster in E. Box/whisker plots indicate interquartile range and 1.5× interquartile range. Statistical tests were determined by paired Wilcoxon test. ****P < 0.0001.

To determine if JAK/STAT signaling was essential for sustaining IFNγ-induced chromatin opening, macrophages were treated with IFNγ for 8 h, washed, and subsequently cultured in regular media or media containing ruxolitinib for another 88 h. Unsupervised k-means clustering revealed three patterns of chromatin behavior after washout when cultured in media alone: increased opening, persistent slight decrease, and abated opening after washout (Fig. 4, A–C; and Data S4). Notably, ruxolitinib treatment after washout reverted chromatin states to baseline, establishing that continued JAK signaling is required for the persistence of chromatin opening following IFNγ stimulation. We observed remarkable similarity between performing this experiment on macrophages from a separate human donor (Fig. S4, A and B; and Data S5).

We asked whether persistent IFNγ-induced transcription factor activity also sustained the expression of ISGs. RNA-sequencing (RNAseq) analysis identified 248 IFNγ-induced genes at 8 h of IFNγ treatment (Data S6), with 51 of these genes retaining at least 90% and 82 genes maintaining 20–90% of their expression following 88 h of washout (Fig. 4, D and E). Ruxolitinib markedly reduced persistent gene expression, with no genes maintaining expression above 90% and only 24 genes retaining expression levels above 20%. To confirm that persistence of gene expression was dependent on IFNγ signaling rather than nonspecific effects of ruxolitinib, we repeated this experiment using an anti-IFNγ neutralizing antibody. Quantitative PCR (qPCR) of two persistent genes, *IRF1* and *IDO1*, demonstrated that anti-IFNγ antibody also significantly reduced expression of these genes after washout (Fig. 4, F and G).

### IFNγ-induced *de novo* enhancers are reversed upon blockade of IFNγ signaling

**Figure 5.**
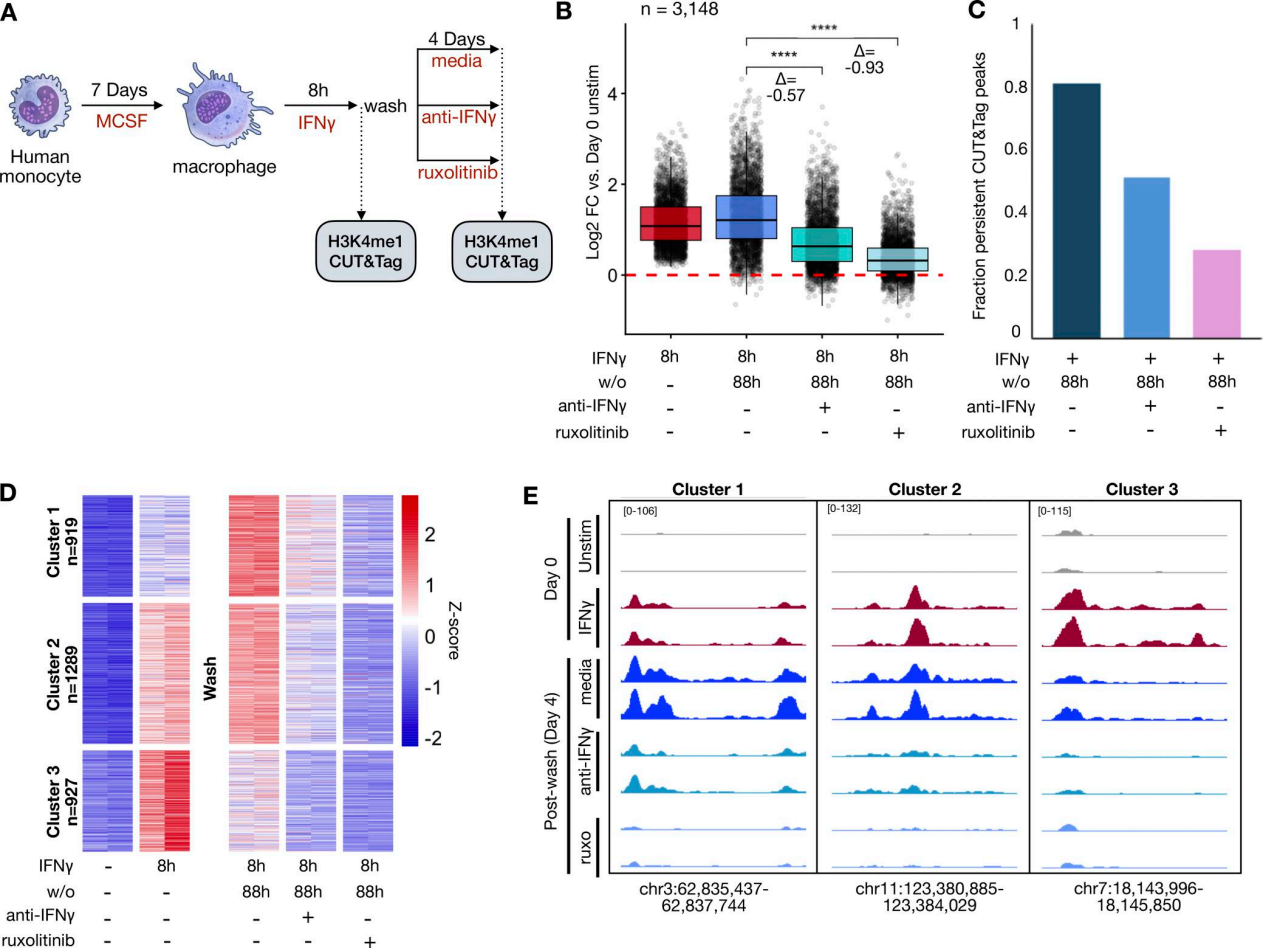
**Durability of IFNγ-induced *de novo* enhancers is dependent on continued JAK/STAT signaling by IFNγ. (A)** Schematic of experimental design: Human macrophages were stimulated with IFNγ (100 ng/ml) for 8 h. Cells were subsequently washed and cultured for an additional 88 h in standard media or media supplemented with 1 µM ruxolitinib or 8 µg/ml anti-IFNγ neutralizing antibody. H3K4me1 CUT&Tag was performed at each time point. **(B)** Boxplot quantifying log2 fold changes of reads within IFNγ-induced H3K4me1 CUT&Tag peaks after 8 h of IFNγ stimulation and after washout for each condition. **(C)** Barplot showing fraction of IFNγ-induced H3K4me1 peaks at 8 h that persist 4 days after washout in each condition. Persistence was defined as L2FC ≥ 0, FDR < 0.01. **(D)**. Heatmap of Z-scored reads within H3K4me1 peaks induced IFNγ (L2FC > 2, FDR < 0.01) after 8 h of stimulation and 4 days after washout for each condition. Clusters were generated by unsupervised k-means clustering. Each column represents a biological replicate from the same human donor. **(E)** Representative genome browser tracks of peaks from each cluster in D. All box/whisker plots indicate interquartile range and 1.5× interquartile range. Statistical tests were determined by paired Wilcoxon test. ***P < 0.001; ****P < 0.0001.

We next asked whether persistent signaling is necessary for maintenance of enhancer marks after IFNγ washout (Fig. 5 A). We found that anti-IFNγ neutralizing antibody and ruxolitinib markedly reduced the persistence of H3K4me1 signals within IFNγ-induced *de novo* enhancers after washout (Fig. 5 B and Data S7). 81.2% of all peaks persisted 88 h after washout when cells were cultured in regular media, compared with 51.4% and 28.2% in media with neutralizing antibody and ruxolitinib, respectively (Fig. 5 C). Unsupervised k-means clustering on the H3K4me1 peaks again revealed three patterns of behavior of peaks after washout: increased reads, unchanged reads, and decreased reads. Addition of neutralizing antibody or ruxolitinib decreased reads in each cluster, demonstrating that the persistence of enhancer marks in each required persistent IFNγ signaling (Fig. 5, D and E). We repeated this experiment on macrophages generated from peripheral blood mononuclear cells (PBMCs) from a separate human donor and saw a remarkably similar pattern of enhancer dependence on continued IFNγ signaling (Fig. S4, C and F; and Data S8).

### IFNγ potentiation of inflammatory gene expression responses days after washout depends on persistent JAK signaling

Macrophages pulsed with IFNγ exhibit potentiated expression of inflammatory genes upon exposure to PAMPs such as LPS (Garrett et al., 2008; Chavez et al., 2025). We asked whether sustained signaling was required for maintaining the potentiated state. To this end, we exposed macrophages for 8 h with IFNγ or vehicle control and cultured them for another 4 days before exposing them to LPS and collecting samples at 1, 3, 6, and 12 h of stimulation for RNAseq (Fig. 5 A). We defined potentiated genes as those that displayed at least a fivefold increase in CPM upon LPS stimulation and at least a twofold greater expression in IFNγ-pre-treated cells compared with PBS at two continuous time points.

**Figure S5.**
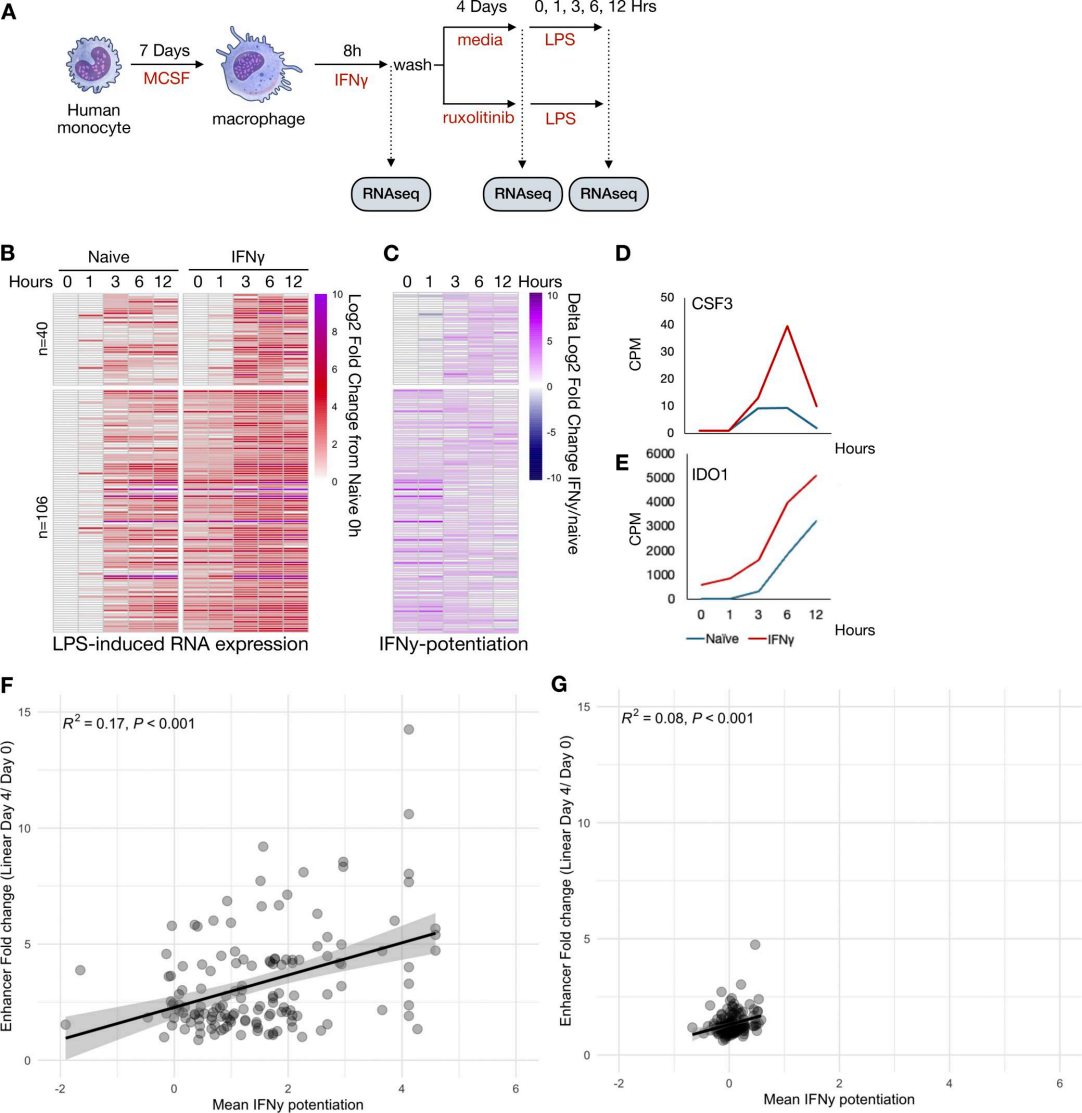
**IFNγ exposed macrophages exhibit potentiated inflammatory gene expression upon LPS restimulation. (A)** Human macrophages were stimulated with IFNγ (100 ng/ml) for 8 h. Cells were subsequently washed and cultured for an additional 88 h in standard media, or media supplemented with 1 µM ruxolitinib, at which time they were stimulated with 10 ng/ml LPS and cultured for an additional 12 h. RNAseq was performed at each time point. **(B)** Heatmap of log2 fold change in reads of LPS genes potentiated by IFNγ before treatment. Log2 fold changes are normalized to PBS-treated controls 88 h after washout prior to LPS stimulation (Naïve 0H). Potentiated genes defined as those reaching fivefold increase in reads after LPS stimulation and at least a twofold greater expression in IFNγ pre-treated cells compared with PBS. Genes are clustered by expression level 88 h after IFNγ washout. The top cluster of genes showed L2FC < 0.5 in IFNγ-treated cells compared with PBS treated; the bottom cluster showed L2FC >0.5 compared with PBS trained. **(C)** Heatmap quantifying extent of IFNγ-induced potentiation. The difference in L2FC for a given genes between PBS and IFNγ treated is quantified for each gene in F. **(D)** Example of CPM for a potentiated gene that showed basal expression equivalent to that of PBS-treated cells: *CSF3*. **(E)** Example of CPM for a potentiated gene that showed basal expression higher than that of PBS-treated cells: *IDO1*. **(F)** IFNγ-induced H3K4me1 CUT&Tag peaks (as defined in Fig. 1) were linked to protein-coding genes within ±20 kb of a gene’s TSS. Promoter-proximal (±1 kb of TSS) peaks were excluded from analysis. Analysis was limited to LPS-induced genes. The mean “IFNγ potentiation” of each gene was calculated (defined as the average of the delta log2 fold change for each gene in C between IFNγ-trained and untrained conditions across all time points). The mean IFNγ potentiation value was plotted against the fold change of the enhancer induced 4 days after IFNγ washout. **(G)** Mean IFNγ potentiation and enhancer fold change in the presence of ruxolitinib as calculated in F.

Using these criteria, we identified 146 LPS-inducible genes potentiated by IFNγ (Fig. S5 B and Data S9). 40 of these genes had basal expression levels that were equivalent to PBS-treated cells (L2FC < 0.5 vs. PBS treated), while 106 exhibited a higher basal expression after the IFNγ pulse. Potentiated genes with a higher basal set point (e.g., *IDO1*) exhibited higher expression over the entire LPS time course, while genes with an unchanged basal set point (e.g., *CSF3*) showed potentiation primarily at later time points of 6 and 12 h (Fig. S5, C and E).

**Figure 6.**
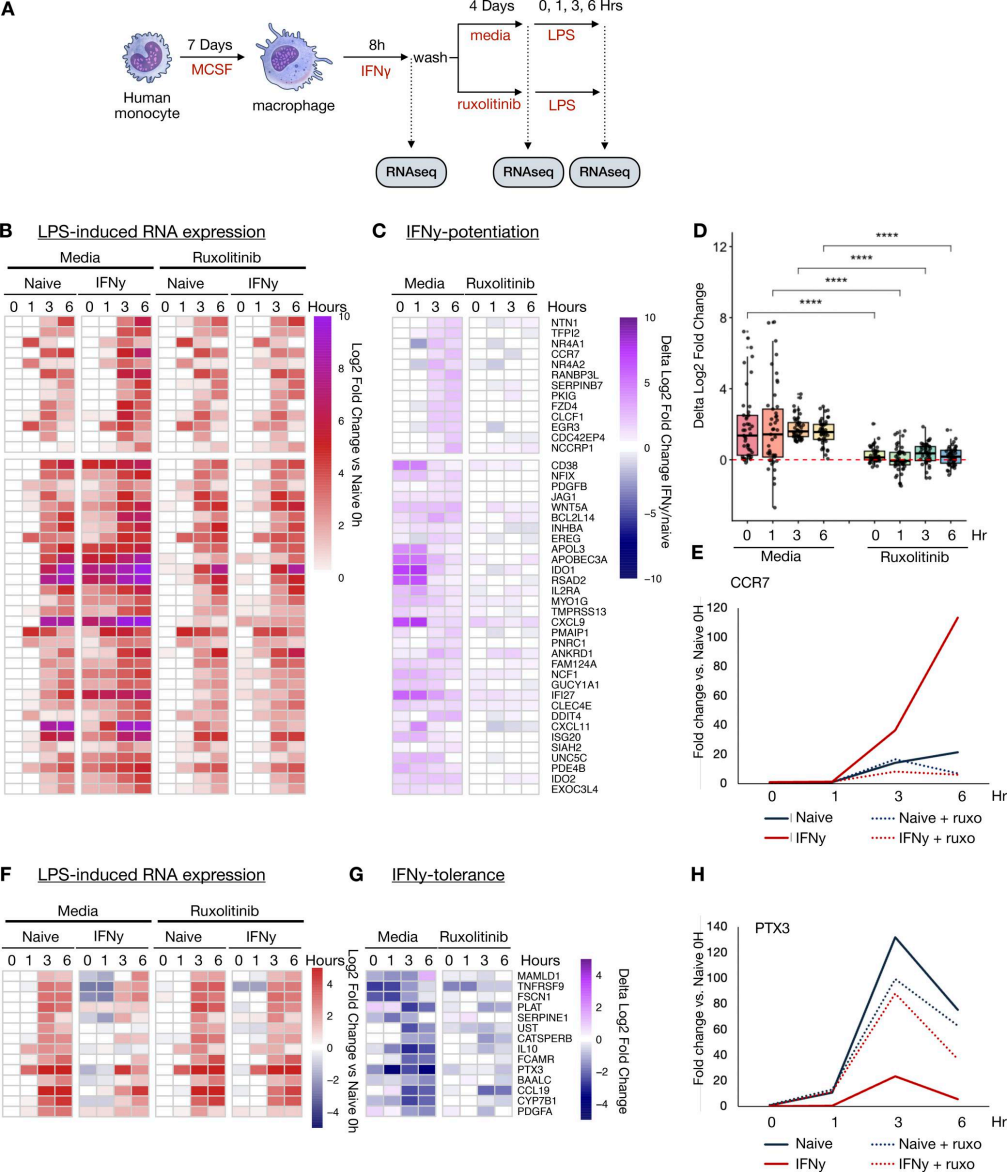
**Sustained JAK/STAT signaling is required for long-term IFNγ-induced potentiated and tolerized gene expression responses. (A)** Human macrophages were stimulated with IFNγ (100 ng/ml) for 8 h. Cells were subsequently washed and cultured for an additional 88 h in standard media, or media supplemented with 1 µM ruxolitinib, at which time they were stimulated with 10 ng/ml LPS and cultured for an additional 6 h. RNAseq was performed at each time point. **(B)** Heatmap of log2 fold change in reads of LPS-induced genes potentiated by IFNγ treatment. Log2 fold changes are normalized to PBS-treated controls 88 h after washout prior to LPS stimulation (Naïve 0H). Potentiated genes are defined as LPS-induced genes reaching at least fourfold increase for macrophages cultured in ruxolitinib and at least a twofold greater expression in IFNγ pre-treated cells compared with PBS in two contiguous time points. Genes are clustered by expression level 88 h after IFNγ washout: the top cluster of genes showed L2FC < 0.5 in IFNγ-treated cells compared with PBS treated; the bottom cluster showed L2FC >0.5 compared with PBS trained. **(C)** Heatmap quantifying extent of IFNγ-induced potentiation. The difference in L2FC for a given gene between PBS and IFNγ treated is quantified for each gene in F. **(D)** Boxplot quantifying difference in L2FC for IFNγ pre-treated and PBS-pre-treated cells at each time point. Box/whisker plots indicate interquartile range and 1.5× interquartile range. Statistical tests were determined by paired Wilcoxon test. ****P < 0.0001. **(E)** Example of fold change for potentiated gene: *CCR7*. **(F)** Heatmap of log2 fold change in reads of LPS-induced genes tolerized by IFNγ. Tolerance is defined as twofold reduction in transcription in two contiguous time points with IFNγ before treatment and at least fourfold induction by LPS in the presence of ruxolitinib. **(G)** Heatmap quantifying extent of IFNγ-induced tolerance as in C. **(H)** Example of fold change for potentiated gene: *PTX3*.

Next, we examined if sustained IFNγ signaling is required for the potentiated LPS response at 4 days after IFNγ washout. To this end, we applied ruxolitinib during the washout phase (Fig. 6 A), but first had to identify LPS-induced genes whose LPS-induction is not blocked by ruxolitinib (Fig. S2). We identified 45 IFNγ-potentiated LPS-inducible genes whose induction is maintained at least fourfold in the presence of ruxolitinib. Of these, 32 showed elevated basal expression levels (L2FC > 0.5) as compared with PBS-treated cells, but 13 did not (Fig. 6 B and Data S10). Remarkably, ruxolitinib almost entirely abolished potentiated expression, leaving a single gene (*ANKRD1*) still meeting potentiation criteria under JAK blockade (Fig. 6, C–E).

In addition to potentiating LPS responses, IFNγ may also induce tolerance in some LPS-induced genes (Kang et al., 2019). We examined tolerized genes in our dataset, defined as genes showing a twofold reduction in transcription in two contiguous time points with IFNγ before treatment and at least fourfold induction by LPS in the presence of ruxolitinib. This analysis identified 14 genes, including well-defined tolerized genes *IL10* and *PTX3* (Fig. 6, F and E; and Data S11). Ruxolitinib treatment abrogated tolerance of all but two genes (*CCL19* and *PLAT*).

Finally, we asked whether the enhancers gained during IFNγ stimulation and lost with ruxolitinib treatment correlated with gene expression potentiated by IFNγ. H3K4me1 CUT&Tag peaks induced by IFNγ were linked to nearby protein-coding genes whose TSS was within 20 kb of the enhancer mark. For each of these genes, we calculated the “mean IFNγ potentiation” score defined as the average of the delta log2 fold change for each gene between IFNγ pre-treated and naïve macrophages upon LPS stimulation. We found a modest but clear direct correlation between the amount of gene potentiation and the gain of linked H3K4me1 marks, while ruxolitinib treatment dramatically diminished both (Fig. 4, E and F).

## Discussion

The described studies revealed that macrophage memory of IFNγ stimulation is not mediated by a chromatin-proximal epigenetic mechanism or altered gene regulatory or signaling network state, but rather depends on persistent signaling by the cytokine itself captured on or near the cell surface. In accord with published literature, we demonstrate that IFNγ treatment of macrophages induces pSTAT1/IRF1-driven *de novo* enhancer formation, which primes macrophages for heightened responsiveness to LPS stimulation (Qiao et al., 2013; Chavez et al., 2025). However, both the potentiated gene expression capacity and enhancer marks are reversible by inhibiting IFNγ signaling with a neutralizing antibody or a JAK inhibitor, demonstrating that sustained signaling is necessary for their durability.

The persistence of IFNγ signaling days after medium washout extends prior studies that reported that IFNγ has the ability to bind directly to cell membranes and the extracellular matrix (ECM) via heparan sulfate and phosphatidyl serine (Lortat-Jacob et al., 1991; Oyler-Yaniv et al., 2017). These interactions may provide a buffering mechanism that spatially constrains the cytokine to the site of infection, thereby preventing cytokine overload and systemic IFNγ-induced toxicity (Kemna et al., 2023). Such ECM-bound IFNγ may be more potent and protected from degradation than soluble cytokine or cytokine in the periphery (Lortat-Jacob et al. 1996; Sadir et al., 1998). Our work indicates that this spatially constrained IFNγ maintains the IFNγ-polarized epigenetic landscape in macrophages, which in turn maintains potentiated gene expression, thereby facilitating long-term innate immune memory.

Prior work with BMDMs has suggested that the polarized transcriptomic state of a cell is reversible after stimulus washout (Liu et al., 2020), yet others have shown persistent pSTAT1 activity and ISG expression after washout in murine melanoma cells (Oyler-Yaniv et al., 2017). We suspect that the persistence of STAT1 phosphorylation and ISG expression is cell type dependent and subject to negative feedback and feed-forward loops that are differentially active in distinct cell types. We emphasize that our results are limited to terminally differentiated macrophages grown *ex vivo* and may not explain findings *in vivo*, where cell progenitors may also be exposed to IFNγ.

At the chromatin level, several studies have demonstrated prolonged cytokine-induced histone modification changes in both human and murine cells (Ostuni et al., 2013; Kamada et al., 2018; Mikulski et al., 2025). Here we show that these epigenetic changes can be reversed with pharmacologic blockade of JAK. In concordance with our results, it has been previously observed that blockade of JAK signaling after IFNγ polarization of monocytes significantly blunts LPS-induced potentiated gene expression (Qiao et al., 2013), demonstrating the need for persistent JAK activity to maintain potentiated gene expression.

Our *in vitro* findings suggest that cytokine-mediated innate immune memory in macrophages is dependent on the surrounding tissue environment rather than being solely intrinsic to the macrophages. Our observations align with recent *in vivo* studies demonstrating that blockade of IFNγ signaling is sufficient to reverse BCG-induced trained immunity in murine models (Lee et al., 2024). It remains to be seen whether this observation extends to other cytokines or pathogen-associated/damage-associated molecular patterns (PAMPs/DAMPs) that can also induce innate immune memory. Notably, several other cytokines and chemokines have also been reported to have an affinity for the ECM or be spatially constrained *in vivo* (Gill et al. 2010; Centofanti et al., 2023; Martinez et al., 2024). We suggest that acute immune activity within a tissue in response to infection or injury may “stain” the tissue with cytokines and that ongoing signaling from these molecules contributes to lasting changes in tissue-resident cells, including macrophages. The observation that the IFNγ-induced memory state is pharmacologically reversible raises the possibility that at least some trained immune states can be pharmacologically erased or modified *in vivo* by blocking cytokine signaling pathways.

Some definitions of innate immune training necessitate that a cell return to a signaling baseline after removal of a stimulus (Netea et al., 2020; Divangahi et al., 2021). This implies a view that innate immune memory is cell intrinsic, residing in chromatin-proximal epigenetic changes or bistable states in gene regulatory or signaling networks. However, we demonstrate that transient exposure to an IFNγ stimulus leads to persistent signaling, resulting in long-term altered responsiveness. We propose that this observation suggests a form of tissue-based immunologic memory, as it is mediated by long-lasting ECM or membrane-sequestered cytokine–receptor interactions that provide continued signaling. As IFNγ-mediated innate immune memory is associated with numerous physiological (Lee et al., 2024) and pathological phenomena (Prevel et al., 2025), locating the memory mechanism to the extracellular domain directs future studies to developing strategies for erasing or reinforcing IFNγ-mediated innate immune training.

## Materials and methods

### Human experimental guidelines and approval

Human blood from deidentified donors was obtained from the University of California, Los Angeles-Charles Drew University center for AIDS research (UCLA-CDU CFAR) Centralized Laboratory Support Core in accordance with UCLA IRB 11-000443. No donors were excluded, and we did not assess whether donors were male or female prior to performing any experiments. Donors were not randomized into groups, and we did not undertake a power analysis because our study does not report statistics between groups or within-group variables.

### Human monocyte/macrophage cell culture

PBMC were isolated from blood by Ficoll (17-1440-03; Cytiva) density centrifugation and cryopreserved in 10% DMSO (D2438; Sigma-Aldrich) in Embryonic Stem Cell FBS (11875-093; Gibco). Monocytes were purified from PBMC using pan-monocyte isolation magnetic beads (130-096-537; Miltenyi). Monocytes were plated on 6-well tissue culture plates at a density of 1.2 × 10^6^ cells/well for 7 days in 3 ml of RPMI (11875-093; Gibco) supplemented with 10% ES FBS (10439-024; Gibco), penicillin-streptomycin (30-002-CI; Corning), L-glutamine (2 mM; 25-005-CI; Corning), and human M-CSF (20 ng/ml; 216-MC-100; R&D Systems). On day 3 of culture M-CSF was replenished to a concentration of 20 ng/ml by adding 60 ng M-CSF in 300 μl medi to each well (assuming that all M-CSF was depleted).

On day 7, macrophages were stimulated with human IFNγ (100 ng/ml; 300-02; PeproTech), LPS (100 ng/ml; L6529-IMG; Sigma-Aldrich), human IFNβ1a (10 ng/ml; 11415-1; PBL Assay Science), or PBS as a vehicle control. Alternate preparations of IFNγ (Fig. S3) were obtained from Acro Biosystems (IFG-H4211) and Sigma-Aldrich (I17001), both were used at 100 ng/ml. The described work involved eight different human donors, a subset of which were used for each of the reported assays. In some conditions, cells were pre-treated with ruxolitinib (1 µM; S1378; Selleck) or cycloheximide (10 µg/ml; C7698; Sigma-Aldrich) for 15 min prior to stimulation. For experiments where cells were washed after stimulation, all media was aspirated from the well, and each well was thoroughly rinsed three times with 3–5 ml HBSS (14-025-134; Thermo Fisher Scientific). Following rinsing, cells were cultured in complete RPMI media for an additional time as indicated in the figures. In some conditions, ruxolitinib (1 µM), CHX (10 µg/ml), anti-IFNγ neutralizing antibody (hifng-mab7-02; InvivoGen at indicated concentrations for Fig. 3 A, or 506532; BioLegend, clone B27, RRID:AB_2801092 for all subsequent experiments at a concentration of 10 µM), or IgG1, κ Isotype control antibody (400166; BioLegend, clone MOPC-21, RRID:AB 11146992 at concentration of 10 µM), was spiked into complete RPMI media.

For RNAseq experiments, macrophages were restimulated 88 h after washout with 100 ng/ml LPS, and samples were collected for up to 12 h after stimulation. In experiments where macrophage supernatants were collected (Fig. 3 D), supernatant was gently aspirated, pelleted at 500 g for 5 min, and cell-free supernatant was frozen at −80°C; supernatant was then quickly thawed at 37°C and immediately applied to fresh macrophages for 1 h. For samples where macrophages were lifted after stimulation (Fig. S3 D), cells were washed as above and then incubated in PBS, 0.5 mM EDTA in PBS, or trypsin (25-052-CV; Corning) for 20 min at 37°C, scraped, and pelleted at 500 g for 5 min. Cells were then replated on a new plate and cultured in media as above for an additional 24 h.

Human A549 airway epithelial cells (RRID:CVCL_0023) were cultured in Dulbecco’s Modified Eagle’s Medium supplemented with 10% fetal bovine serum (FB-11; Omega Scientific), penicillin-streptomycin (30-002-CI; Corning), and L-glutamine (2 mM; 25-005-CI; Corning). Cells were stimulated with IFNγ and washed as above for macrophages.

### CUT&Tag libraries and sequencing

Stimulated and control macrophages were lifted from plates with 0.5 mM EDTA in PBS and gentle scraping. Nuclear isolation and tagmentation were performed on 100,000 cells per manufacturer protocol as previously described (Kaya-Okur et al., 2019) with the CUTANA CUT&Tag pAG-Tn5 enzyme (15-1017; EpiCypher) with the notable exception that a cocktail of 1 mM dithiothreitol, 0.5 mM (D0632; Sigma-Aldrich), Phenylmethanesulfonyl fluoride (206-350-2; Sigma-Aldrich), 4 µg/ml Leupeptin (L9783; Sigma-Aldrich), 1 µM Pepstatin A (P5318; Sigma-Aldrich), and 0.01 trypsin inibitory units(TIU)/ml Aprotinin (A1153; Sigma-Aldrich) substituted the Roche Protease inhibitor cocktail where necessary. Anti-H3K4me1 antibody (ab8895; Abcam, RRID:AB_306847) was used at a dilution of 1:20 as the primary antibody; guinea pig anti-rabbit antibody (ABIN101961; Abcam, RRID:AB_10775589) was used at a dilution of 1:100 as secondary antibody. Libraries were sequenced with paired-end 50 bp reads on an Illumina NovaSeq X Plus. Each library was downsampled to 30 million reads using Seqtk with option -s 100. Low-quality reads were trimmed (cutoff q = 20), and adapter sequences were removed with Cutadapt (Martin, 2011). Reads were aligned to the human hg38 genome using bowtie2 (RRID:SCR_016368) (Langmead and Salzberg, 2012) with default parameters except for the very-sensitive and nondeterministic options. Aligned reads were filtered based on mapping score (MAPQ ≥ 30) with Samtools (RRID:SCR_002105). Duplicated reads were removed with Picard MarkDuplicates (RRID:SCR_006525). Genome browser tracks were generated using the bamCoverage function in deepTools (RRID:SCR_016366) (Ramírez et al., 2016) with the following options: --binSize 10 --smoothLength 30 --normalizeUsing RPGC --effectiveGenomeSize 2913022398. MACS3 (RRID:SCR_013291) (Zhang et al., 2008) was used to identify peaks within CUT&Tag using standard options except -f BAMPE and -q 0.01. We generated a peak file for every condition and then generated a combined peak file across all conditions for each human subject (including all unstimulated and stimulated conditions). These genomic loci were used to generate a counts table using deepTools multiBamSummary for subsequent analysis. CUT&Tag reads are deposited in the GEO database with accession number GSE294916.

### ATAC libraries and sequencing

Stimulated and control macrophages were lifted as described above for CUT&Tag. Nuclear isolation and tagmentation reaction were performed as previously described (Cheng et al., 2021). Briefly, 50,000 cells were used to prepare nuclei in cold lysis buffer (10 mM Tris-HCl, pH 7.5, 3 mM MgCl2, 10 mM NaCl, and 0.1% IGEPAL CA-630). Nuclei were pelleted by centrifugation at 500 *g* for 10 min and suspended in transposase reaction mixture consisting of 25 μl 2× TD Buffer, 2.5 μl TD enzyme (20034197; Illumina), and 22.5 μl water. The transposase reaction was performed at 37°C for 30 min in a thermomixer shaker at 800 rpm. Libraries were prepared with the Nextera DNA library preparation kit and sequenced on an Illumina NovaSeq X Plus. Reads were processed and aligned to the human hg38 genome as above for CUT&Tag. ATAC reads are deposited in the GEO database with accession number GSE294915.

### ATACseq and CUT&Tag analysis

Peaks were first filtered to select only those that were in the top 50th percentile of reads in any condition during acute stimulation (unstimulated, 8 h IFNγ, or 8 h LPS conditions). Pseudocounts were set at the first percentile of CPM for each condition. Differential peaks were identified using edgeR (RRID:SCR_012802) (Robinson et al. 2010) applying a cutoff of FDR < 0.01 and L2FC > 2 compared with unstimulated conditions. Motif analysis to detect top-enriched known motifs was performed with the findMotifsGenome function in the HOMER suite (RRID:SCR_010881) (Heinz et al., 2010) using all detected peaks from each condition in each human subject as background. Genome browser tracks were visualized with IGV (RRID:SCR_011793) (Robinson et al., 2011) using group auto scale across all conditions for each experiment (ATAC and CUT&Tag were group autoscaled separately).

### TOBIAS transcription factor binding inference

ATAC peaks containing HOMER motifs, IRF1 or STAT1 motifs in IFNγ-induced peaks, and NFκB-p65-Rel or IRF1 motifs in LPS-induced peaks were identified using the annotatePeaks.pl function from the HOMER suite (RRID:SCR_010881) (version 4.11). Peaks were filtered and categorized in R by the presence of each motif within the peak within IFNγ and LPS-induced peaks. TOBIAS (https://github.com/loosolab/TOBIAS; Bentsen et al., 2020) (version 0.17.1) was used to identify regions where transcription factor binding was predicted for each condition. ATACorrect, ScoreBigwig, and BINDetect were run with standard options in sequential order for each condition. The input files were bed files of IFNγ and LPS-induced peaks and BAM files of aligned reads for each condition. The above HOMER IRF1, STAT1, and NFκB-p65 motifs were used as input motifs for BINDetect. BINDetect output was used to quantify predicted bound and unbound peaks.

### Immunoblotting

Macrophages were lysed directly on cell culture plates using 2× Laemmli buffer (120 mM Tris-Cl, pH = 6.8, 20% glycerol, 4% SDS, 0.05% β-mercaptoethanol, and 0.02% bromophenol blue), boiled at 95°C, and then stored at −80°C. Equal amounts of protein were loaded in 10% Tris-Glycine gels (Mini-PROTEAN TGX Gels, #456-1036; Bio-Rad) separated by molecular weight by electrophoresis at 150 V for 1 h. Protein was transferred to PVDF membranes at 100 V for 1 h. Membranes were incubated in 5% bovine serum albumin (BSA, #A9647; Sigma-Aldrich) for 1 h, then incubated in primary antibodies. The following primary antibodies were used: pSTAT1 pY701.4A (#136229; Santa Cruz Biotechnology, RRID:AB_2019074) diluted at 1:10,000, IRF1 D5E4 (#8478; Cell Signaling Technologies, RRID:AB_10949108) diluted at 1:1,000, β-tubulin TUB2.1 (T5201; Sigma-Aldrich, RRID:AB_609915) diluted at 1:10,000, and GAPDH H-12 (#166574; Santa Cruz Biotechnology, RRID:AB_2107296) diluted at 1:10,000. Incubation for pSTAT1 and IRF1 was performed overnight at 4°C, and incubation for β-tubulin and GAPDH was performed for 1 h at room temperature. Incubation with secondary HRP-conjugated antibodies (anti-mouse IgG, #7076; Cell Signaling Technologies, RRID:AB_330924; anti-rabbit IgG, #7074; Cell Signaling Technologies, RRID:AB_2099233) was performed for 1 h at room temperature. Protein was visualized by application of SuperSignal West Pico PLUS Chemiluminescent Substrate (#34580; Thermo Fisher Scientific) and exposure on a Bio-Rad ChemiDoc MP Imaging System, using Bio-Rad Image Lab software (version 5.2). Immunoblots were quantified using the Fiji package in ImageJ (https://imagej.net/software/fiji/, RRID:SCR_002285).

### RNAseq sample preparation and analysis

Macrophages were treated as described above. Cells were lysed directly on the plate with Qiagen RLT buffer. RNA was extracted using Qiagen Qiashredder (79656; Qiagen) and RNEasy mini kit (74104; Qiagen) according to the manufacturer protocol. Library preparation and sequencing were performed by BGI using the DNBseq platform on an MGI T7 machine. Low-quality reads were trimmed (cutoff q = 20), and adapter sequences were removed with cutadapt. Reads were aligned to the hg38 human genome using STAR (Dobin et al., 2013) with the following options: --outSAMunmapped Within, --outSAMtype BAM SortedByCoordinate, --outFilterType BySJout, --outFilterMultimapNmax 20, --alignSJoverhangMin 8, --alignSJDBoverhangMin 1, --outFilterMismatchNmax 999, --alignIntronMin 20, --alignIntronMax 1000000, --alignMatesGapMax 1000000, --outFilterMismatchNoverLmax 0.04, and --seedSearchStartLmax 30. Aligned reads were filtered based on mapping score (MAPQ ≥ 30) by Samtools (RRID:SCR_002105). Counts for each gene were generated using featureCounts (Liao et al. 2014). Counts were normalized by CPM, pseudocount was set at CPM of 1. Analysis of gene expression was limited to protein coding genes. RNAseq reads are deposited in the GEO database under accession number GSE294918.

### Gene expression analysis by RT-qPCR

Macrophages were lysed directly on tissue culture plates, and RNA was collected and purified using RNeasy Mini Kit (#74106; Qiagen). Equal amounts of RNA were reverse transcribed into cDNA using LunaScript RT SuperMix Kit (#E3010; New England Biolabs). qPCR was performed using the Luna Universal qPCR Master Mix (#M3003; New England Biolabs) with 0.25 μM each of forward and reverse primers for target genes: IDO1 (5′-TTC​AGT​GCT​TTG​ACG​TCC​TG-3′; 5′-TGG​AGG​AAC​TGA​GCA​GCA​T-3′), IRF1 (5′-GCT​GGG​ACA​TCA​ACA​AGG​AT-3′; 5′-CTT​CCA​CGT​CTT​GGG​ATC​TG-3′), and HPRT1 (5′-AGG​ACT​GAA​CGT​CTT​GCT​CG-3′; 5′-ATC​CAA​CAC​TTC​GTG​GGG​TC-3′). CT values for target genes were normalized to internal HPRT controls.

### Online supplemental material

Fig. S1 presents data comparing the results of ATACseq and H3K4me1 CUT&Tag. Fig. S2 shows the dose titration of ruxolitinib in human macrophages, and its ability to block STAT1/2 phosphorylation induced by IFNγ and LPS. Fig. S3 provides additional data supporting the notion that persistent pSTAT1 in human macrophages is mediated by IFNγ sequestered at the cell surface. Fig. S4 repeats ATAC and CUT&Tag experiments with a second human subject to show the generalizability of the results. Fig. S5 presents RNAseq results demonstrating the dynamics of IFNγ-mediated potentiation of LPS-induced gene expression. Supplementary data files present the raw CPM counts of next-generation sequencing data used to generate figures in this publication as follows: Fig. 1 B (Data S1), Fig. 1 G (Data S2), Fig. 2 B (Data S3), Fig. 4 B (Data S4), Fig. S4 A (Data S5), Fig. 4 D (Data S6), Fig. 5, B–D (Data S7), Fig. S4 C (Data S8), Fig. S5 B (Data S9), Fig. 6 B (Data S10), and Fig. 6 F (Data S11).

## Supplementary Material

Data S1 shows the raw CPM counts of next-generation sequencing data used to generate Fig. 1 B.

Data S2 shows the raw CPM counts of next-generation sequencing data used to generate Fig. 1 G.

Data S3 shows the raw CPM counts of next-generation sequencing data used to generate Fig. 2 B.

Data S4 shows the raw CPM counts of next-generation sequencing data used to generate Fig. 4 B.

Data S5 shows the raw CPM counts of next-generation sequencing data used to generate Fig. S4 A.

Data S6 shows the raw CPM counts of next-generation sequencing data used to generate Fig. 4 D.

Data S7 shows the raw CPM counts of next-generation sequencing data used to generate Fig. 5, B–D.

Data S8 shows the raw CPM counts of next-generation sequencing data used to generate Fig. S4 C.

Data S9 shows the raw CPM counts of next-generation sequencing data used to generate Fig. S5 B.

Data S10 shows the raw CPM counts of next-generation sequencing data used to generate Fig. 6 B.

Data S11 shows the raw CPM counts of next-generation sequencing data used to generate Fig. 6 F.

SourceData F2 is the source file for Fig. 2.

SourceData F3 is the source file for Fig. 3.

SourceData FS2 is the source file for Fig. S2.

SourceData FS3 is the source file for Fig. S3.

## Data Availability

Primary sequencing data for RNAseq, ATACseq, and CUT&Tag results are publicly available via the National Center for Biotechnology Information Gene Expression Omnibus under accession numbers GSE294918, GSE294915, and GSE294916, respectively.
