# Supplementary figures and images for: IFNγ-induced memory in human macrophages is sustained by the durability of cytokine signaling itself
Source: J Exp Med. 2026 Feb 18;223(4):e20250976. doi: 10.1084/jem.20250976 (PMC12915527; doi:10.1084/jem.20250976)

F2G\_pSTAT1

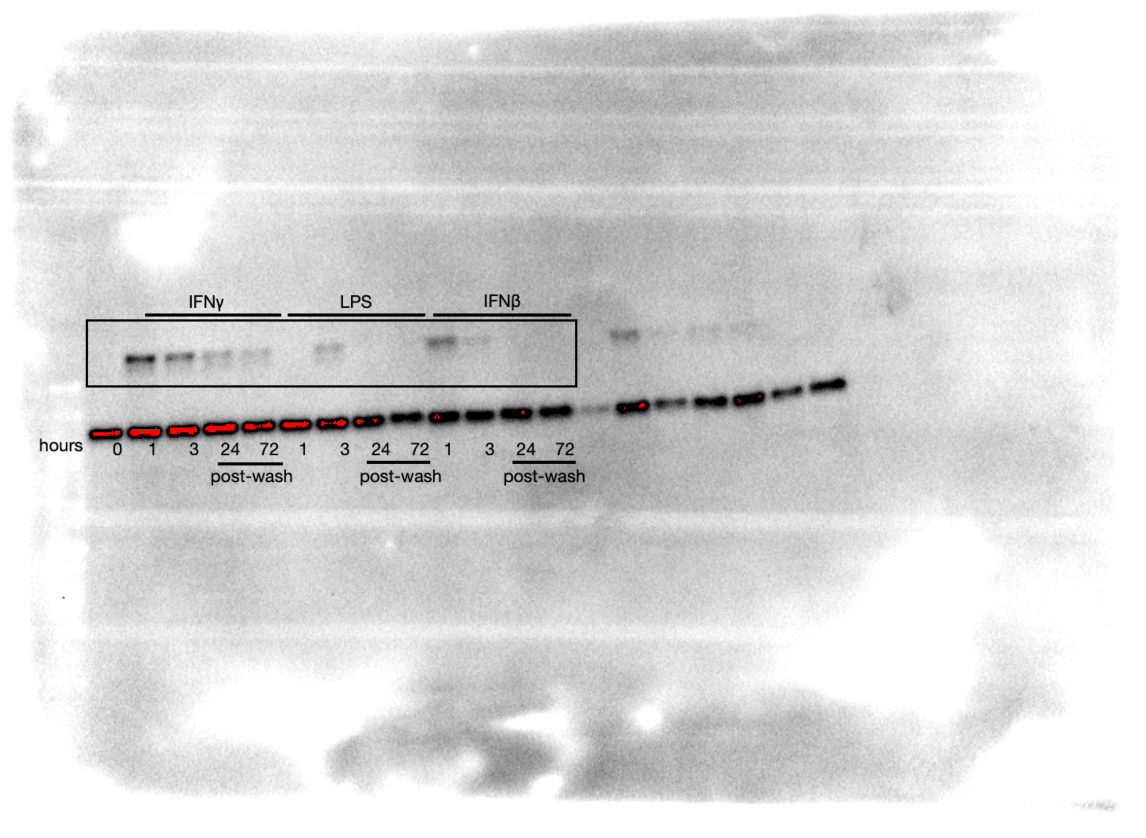

F2G\_tubulin

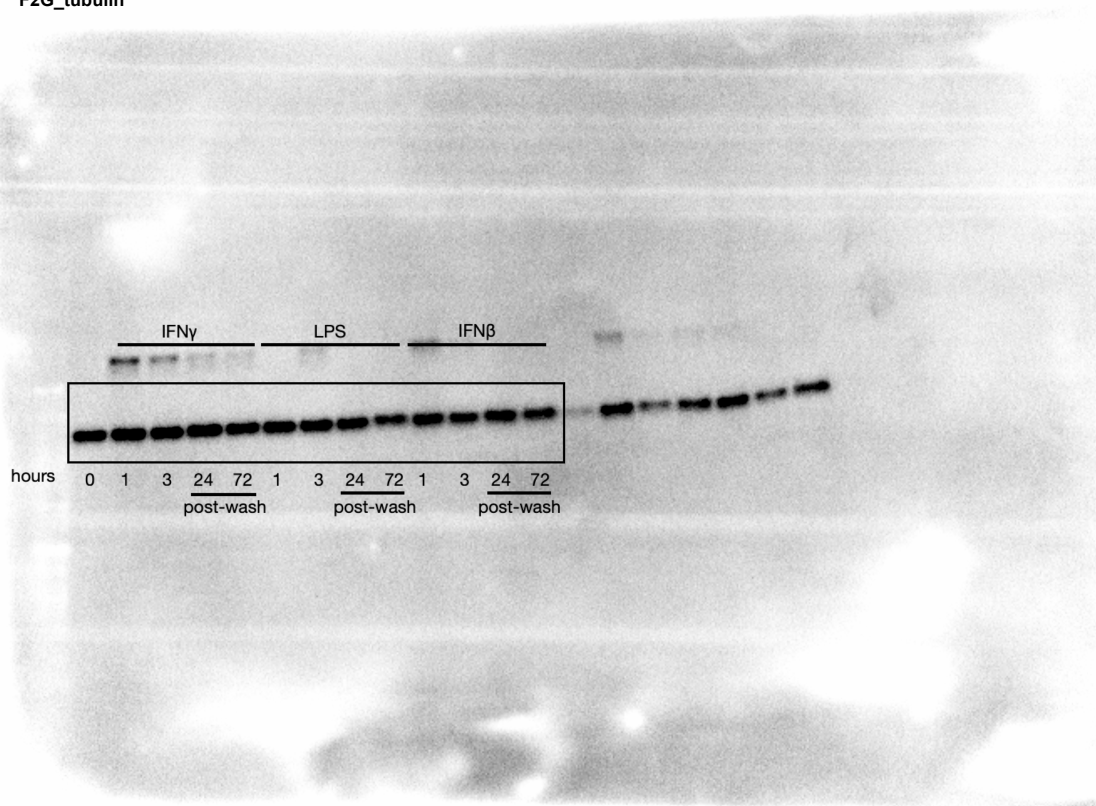

Supplement: SourceData F2 — is the source file for Fig. 2. [file jem_20250976_sourcedataf2.pdf]

FS2\_pSTAT1

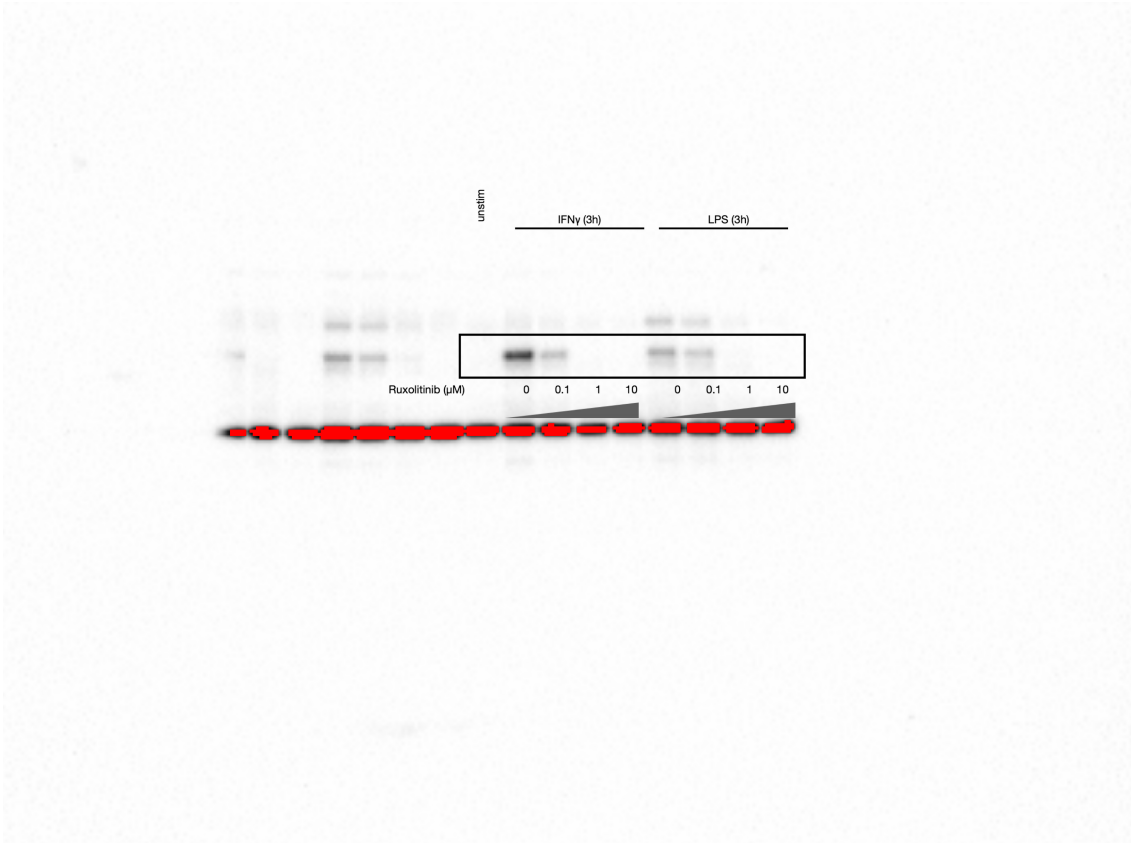

FS2\_pSTAT2

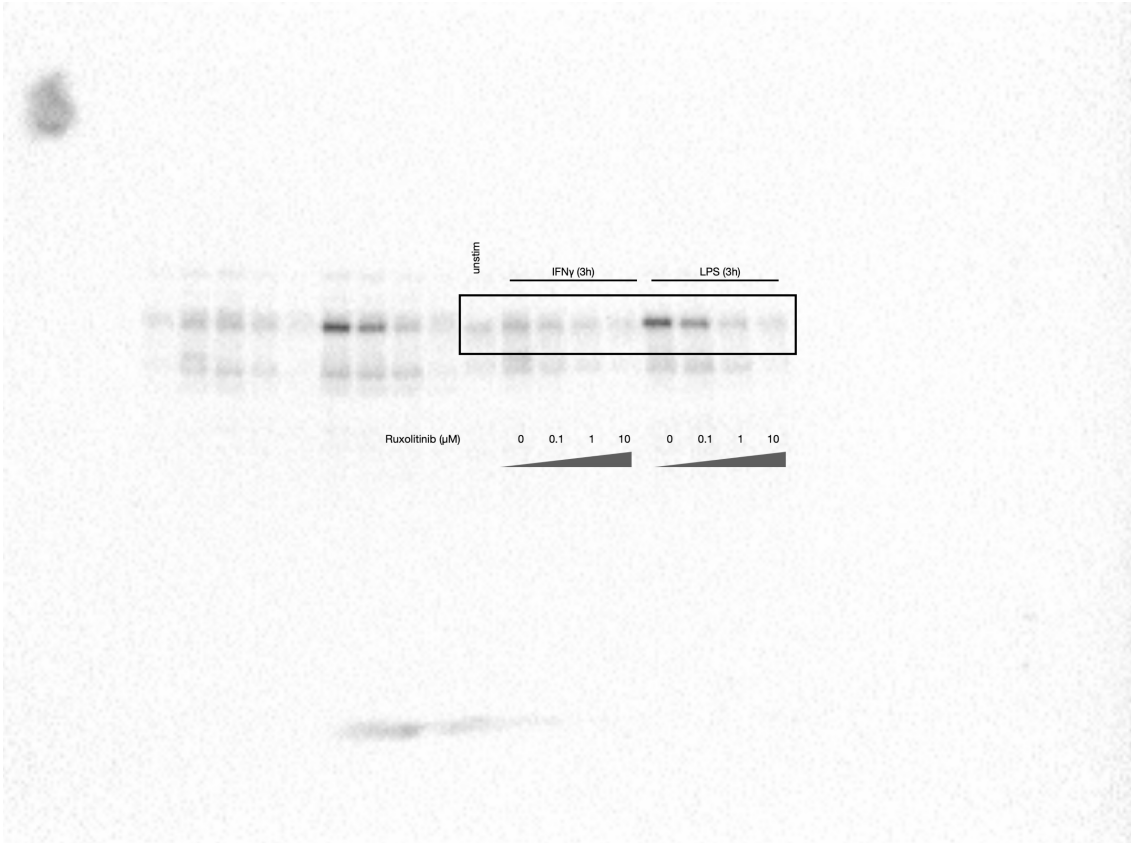

FS2\_tubulin

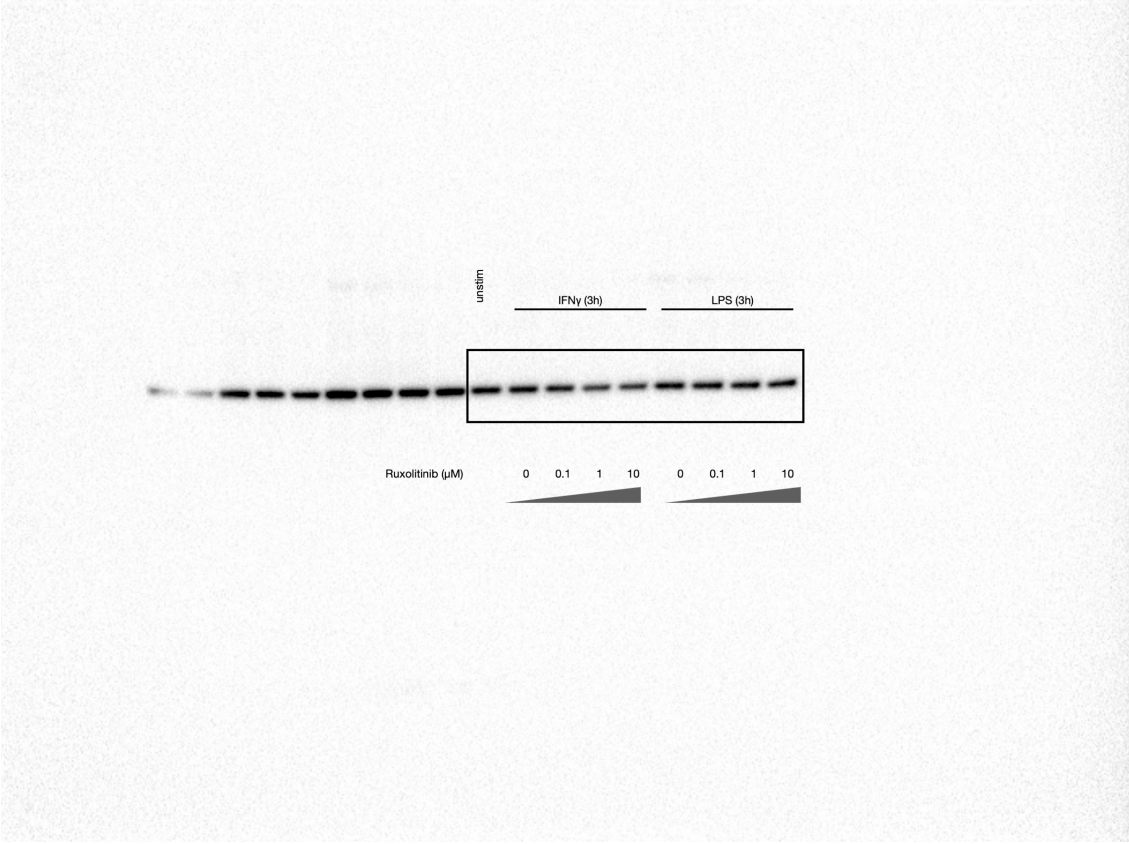

Supplement: SourceData FS2 — is the source file for Fig. S2. [file jem_20250976_sourcedatafs2.pdf]

FS3B\_pSTAT1\_tubulin

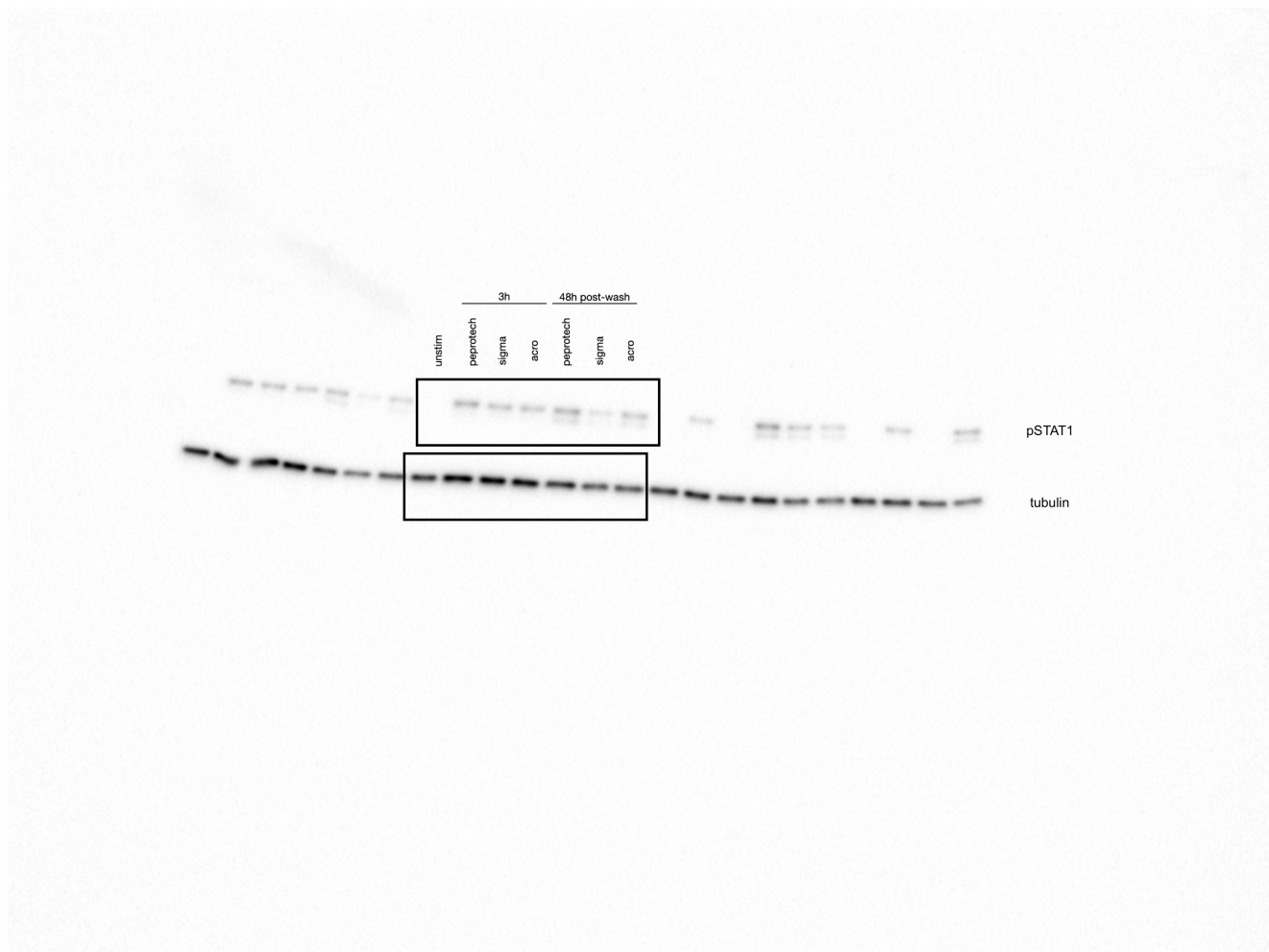

FS3D\_pSTAT1

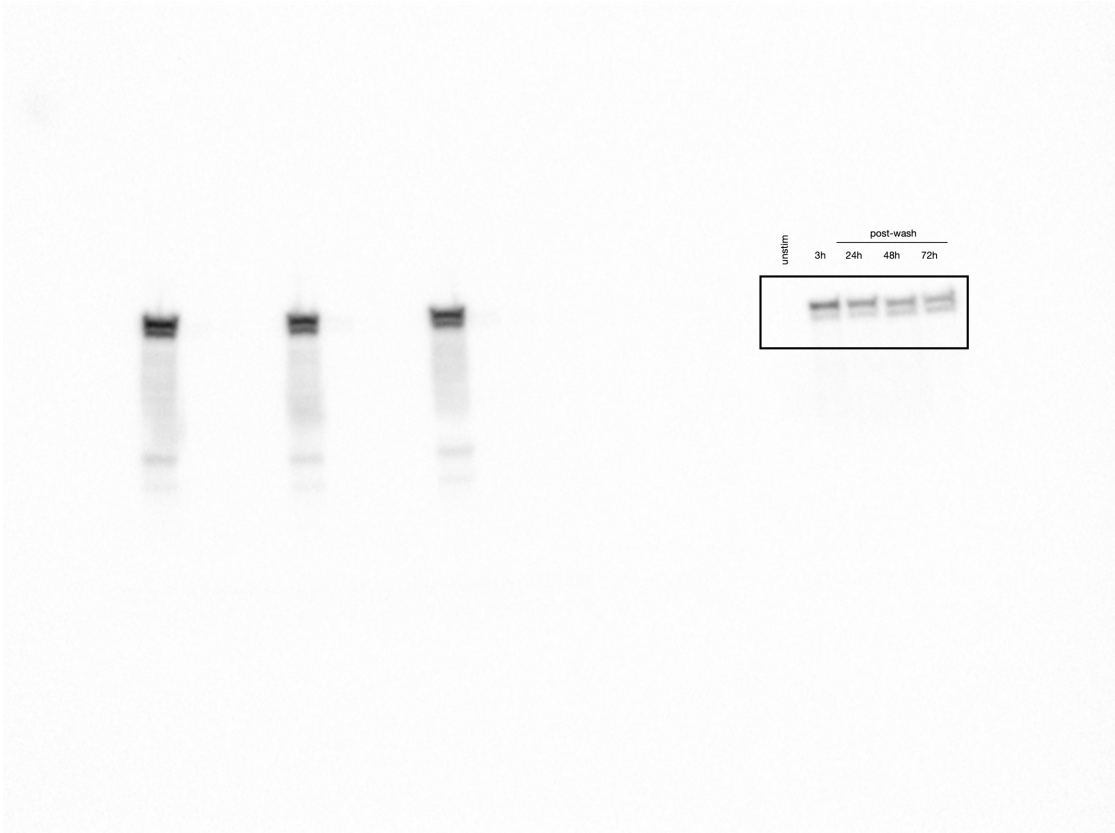

FS3D\_tubulin

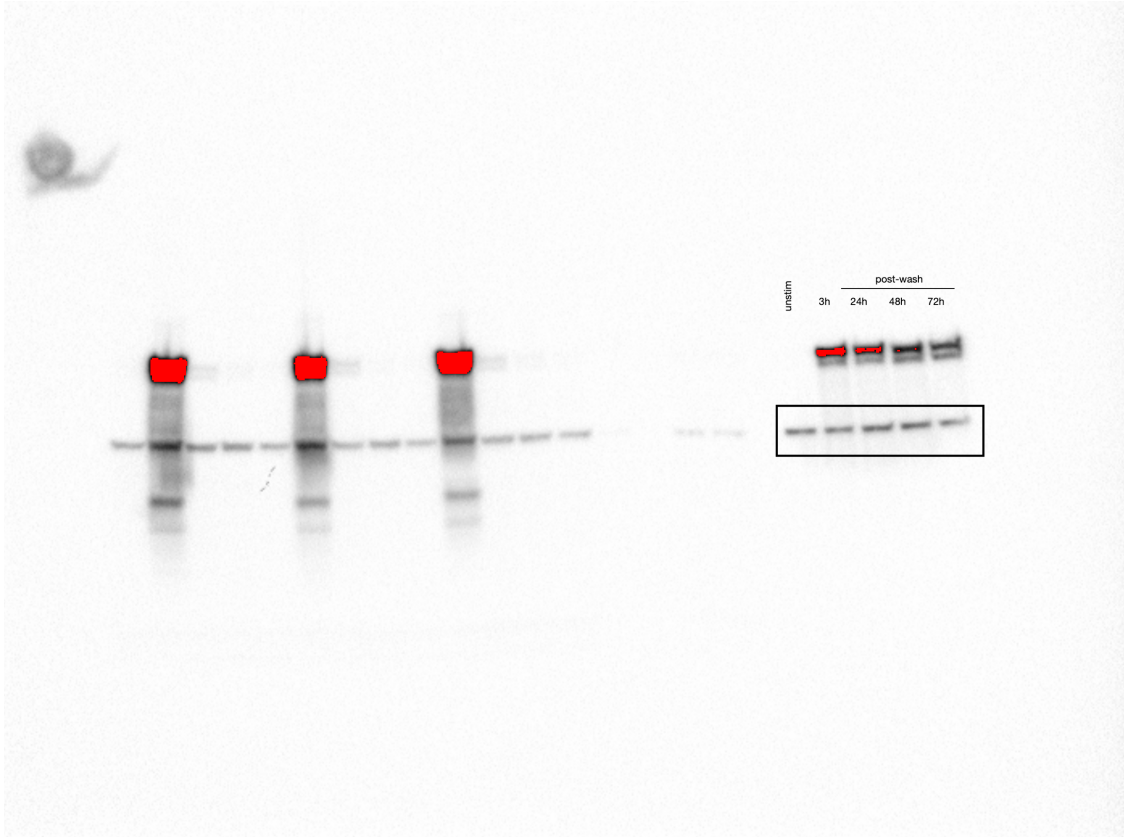

FS3F\_pSTAT1

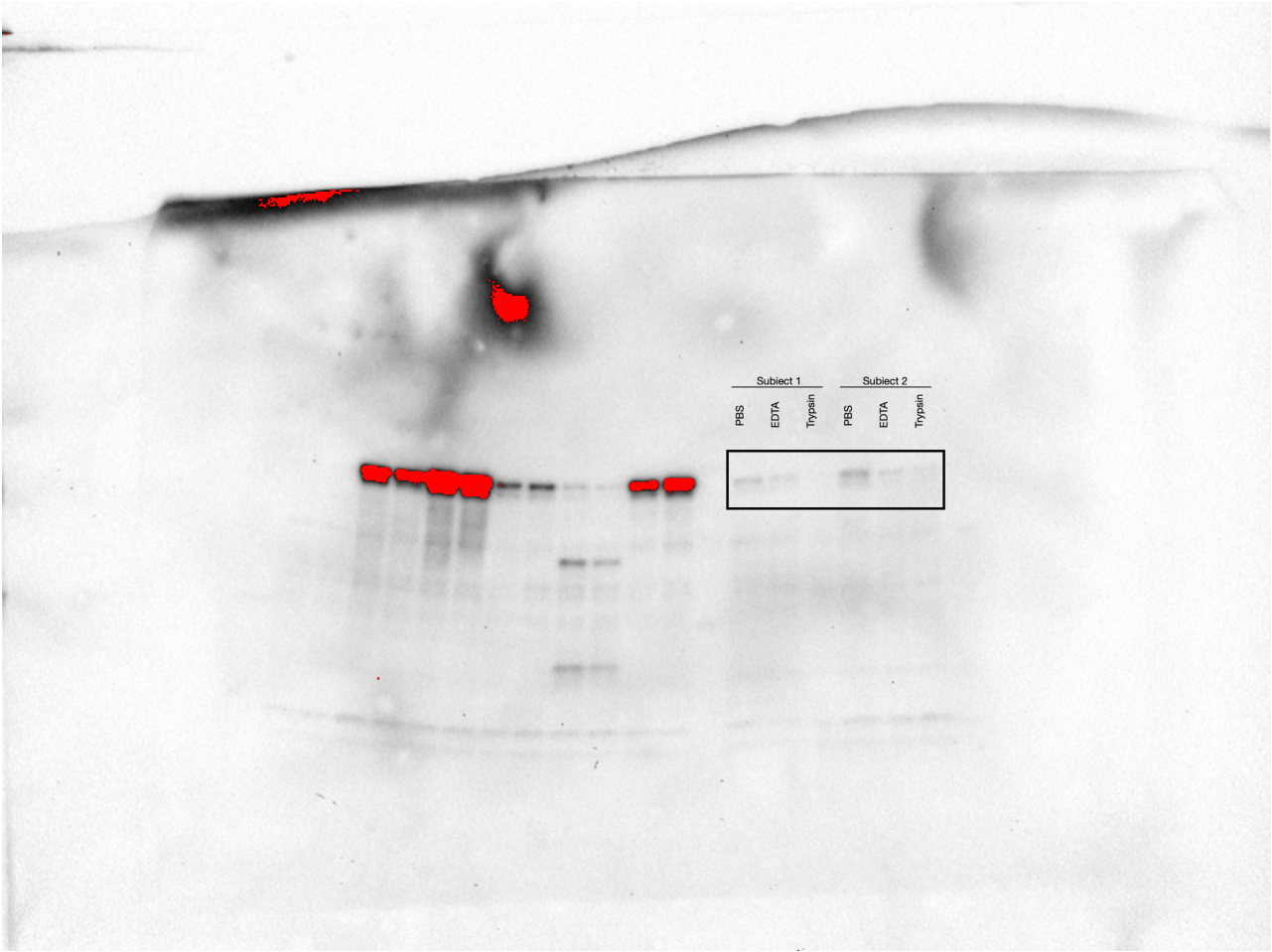

FS3F\_GAPDH

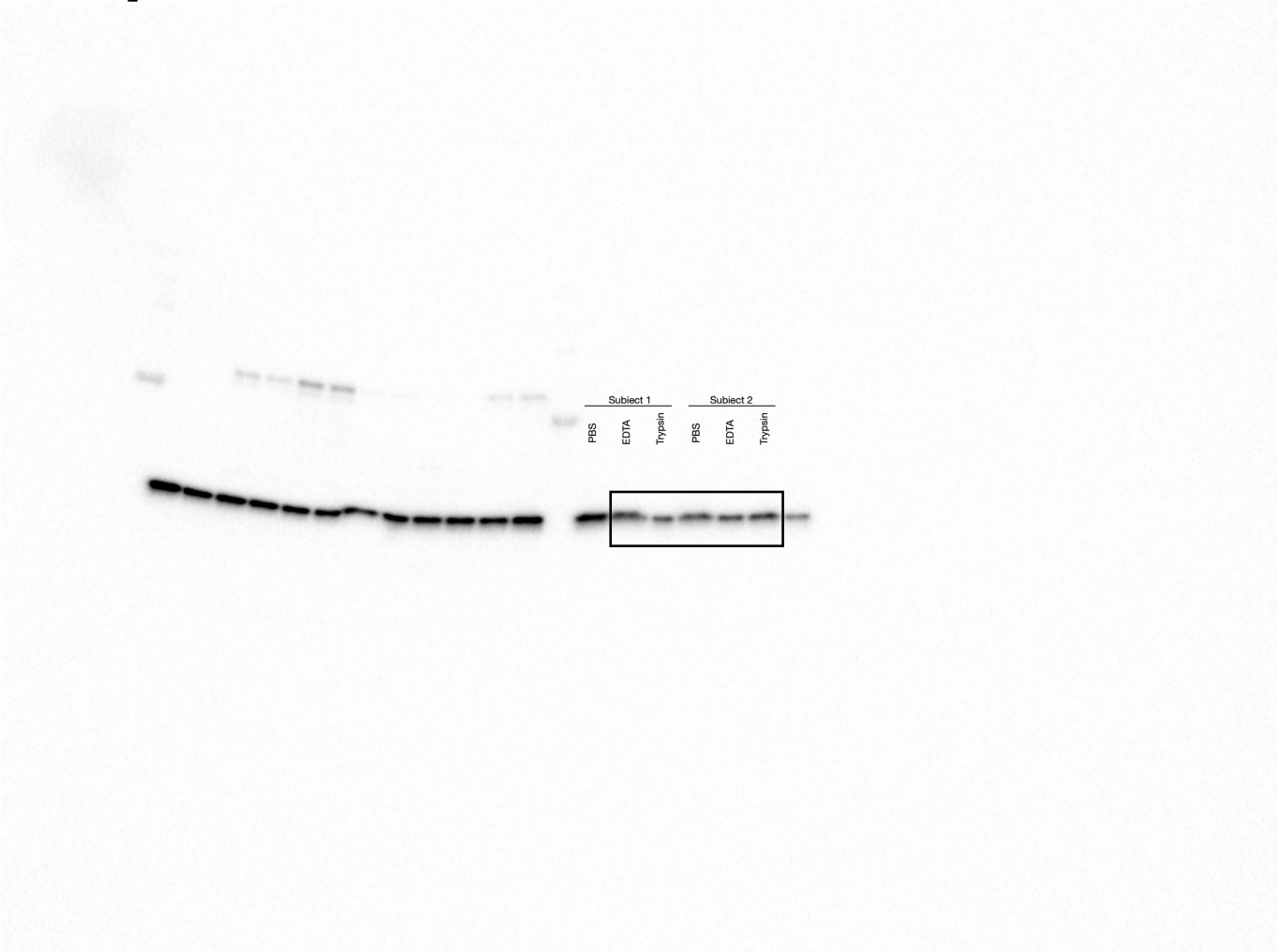

Supplement: SourceData FS3 — is the source file for Fig. S3. [file jem_20250976_sourcedatafs3.pdf]
