# Supplementary material for: IFNγ-induced memory in human macrophages is sustained by the durability of cytokine signaling itself
Source: J Exp Med. 2026 Feb 18;223(4):e20250976. doi: 10.1084/jem.20250976 (PMC12915527; doi:10.1084/jem.20250976)

F3A_pSTAT1

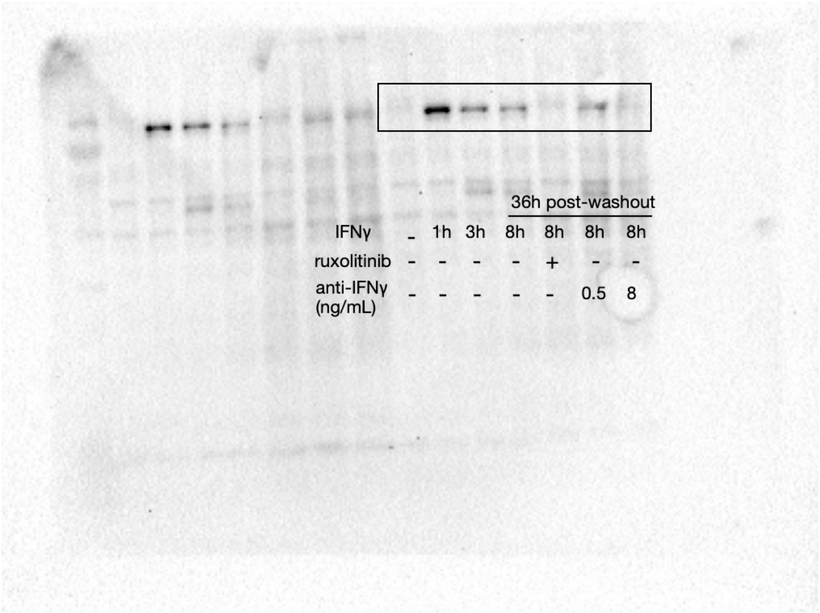
36h post-washout
IFNγ - 1h 3h 8h 8h 8h 8h
ruxolitinib - - - - + - -
anti-IFNγ (ng/mL) - - - - - 0.5 8

F3A_IRF1

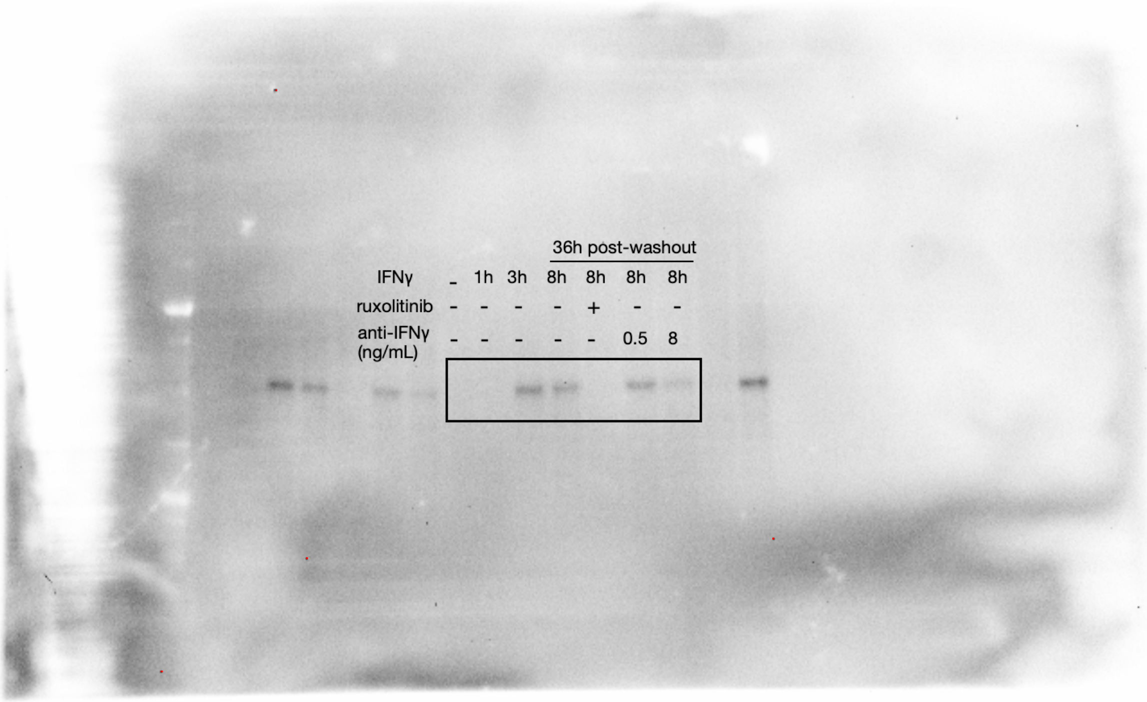
36h post-washout
IFNγ - 1h 3h 8h 8h 8h 8h
ruxolitinib - - - - + - -
anti-IFNγ (ng/mL) - - - - - 0.5 8

F3A_tubulin

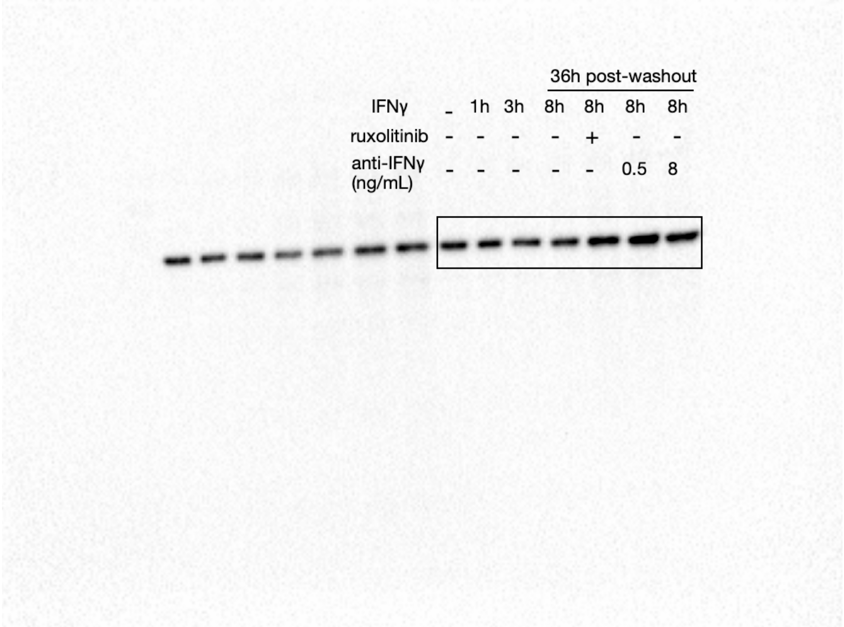
36h post-washout
IFNγ - 1h 3h 8h 8h 8h 8h
ruxolitinib - - - - + - -
anti-IFNγ (ng/mL) - - - - - 0.5 8

F3B_pSTAT1

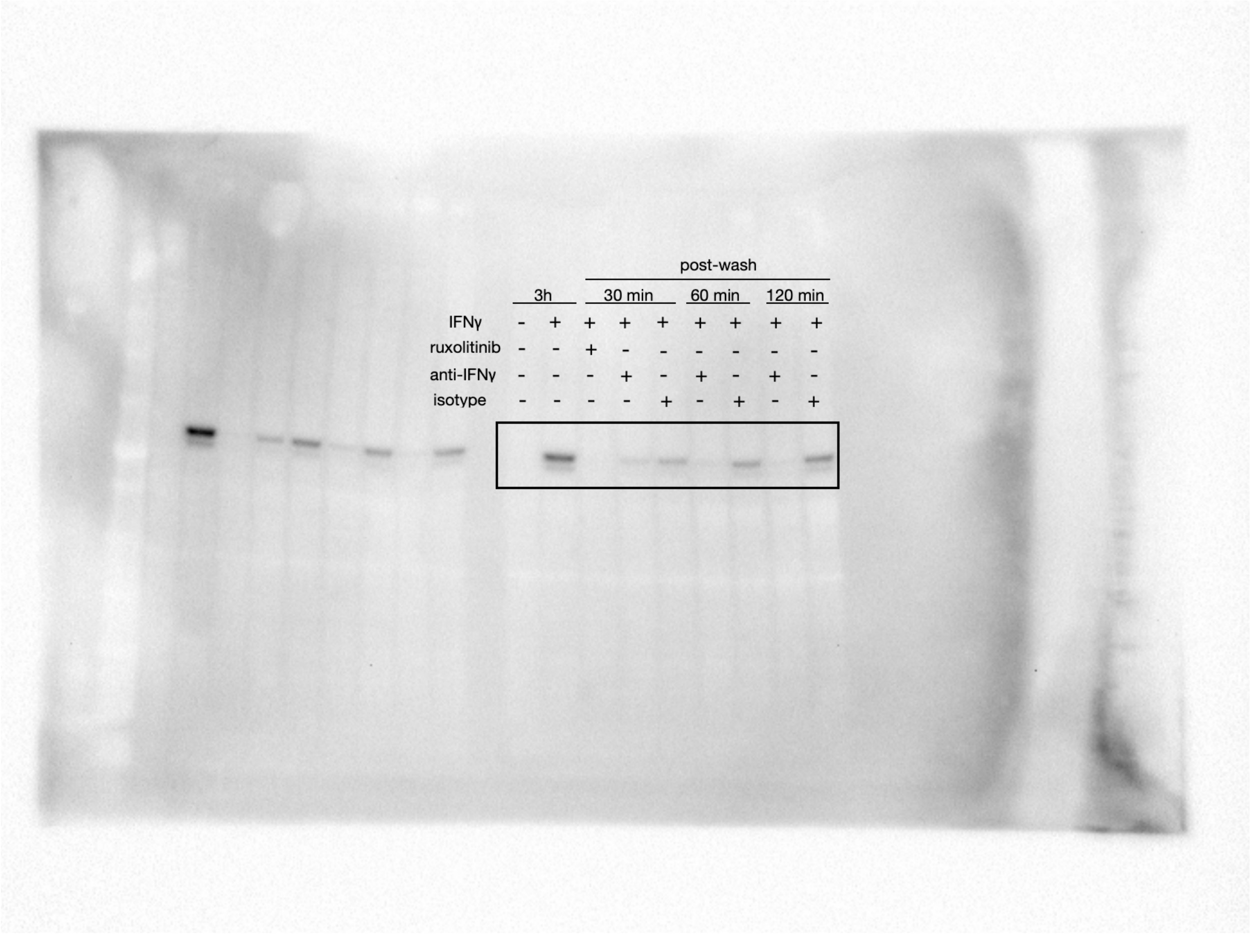

post-wash
3h
30 min
60 min
120 min
IFNγ - + + + + + + + +
ruxolitinib - - + - - - - - -
anti-IFNγ - - - + - + - + -
isotype - - - - + - + - +

F3B_tubulin

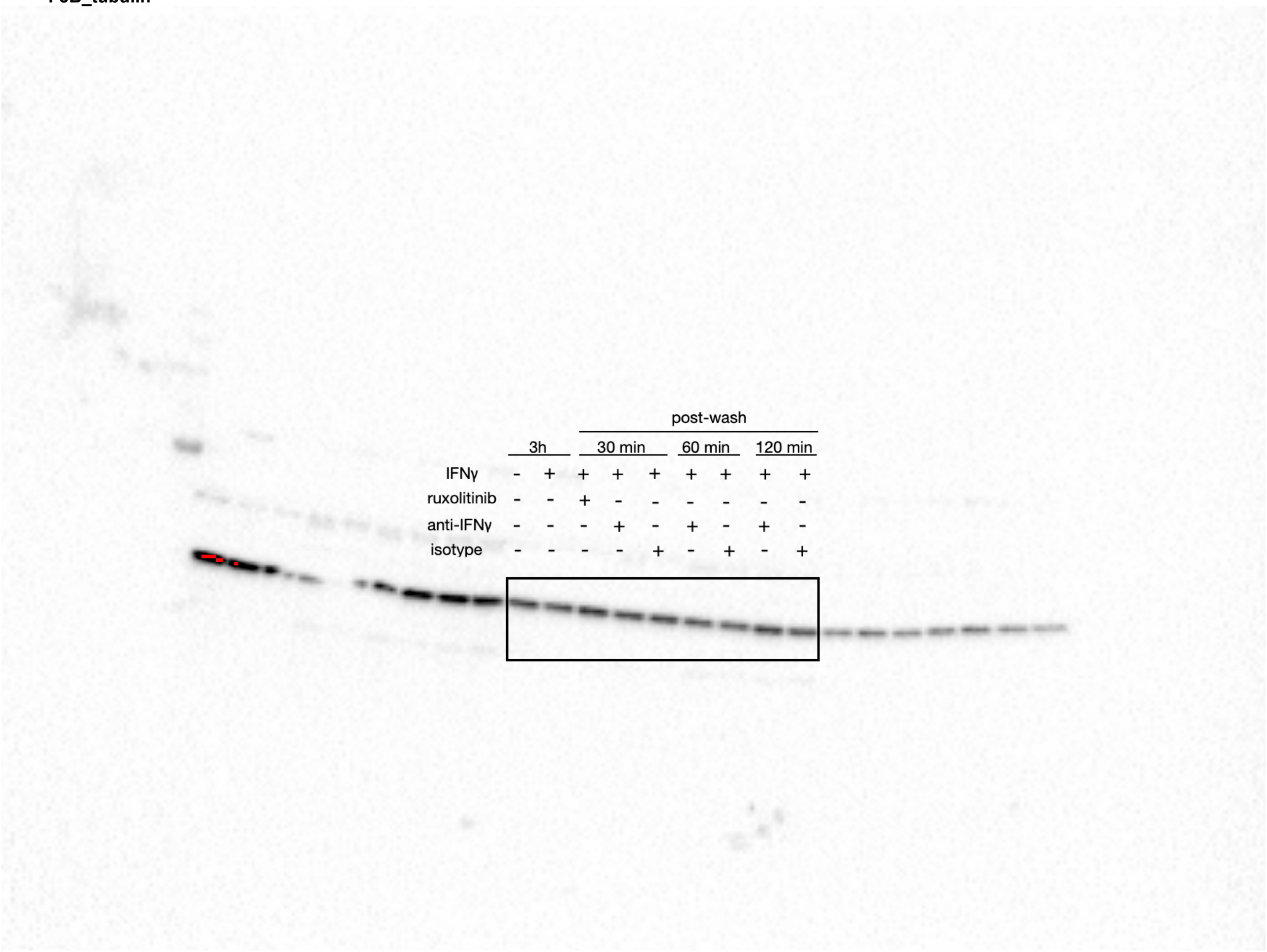

post-wash
3h
30 min
60 min
120 min
IFNγ - + + + + + + + +
ruxolitinib - - + - - - - - -
anti-IFNγ - - - + - + - + -
isotype - - - - + - + - +

F3D_pSTAT1

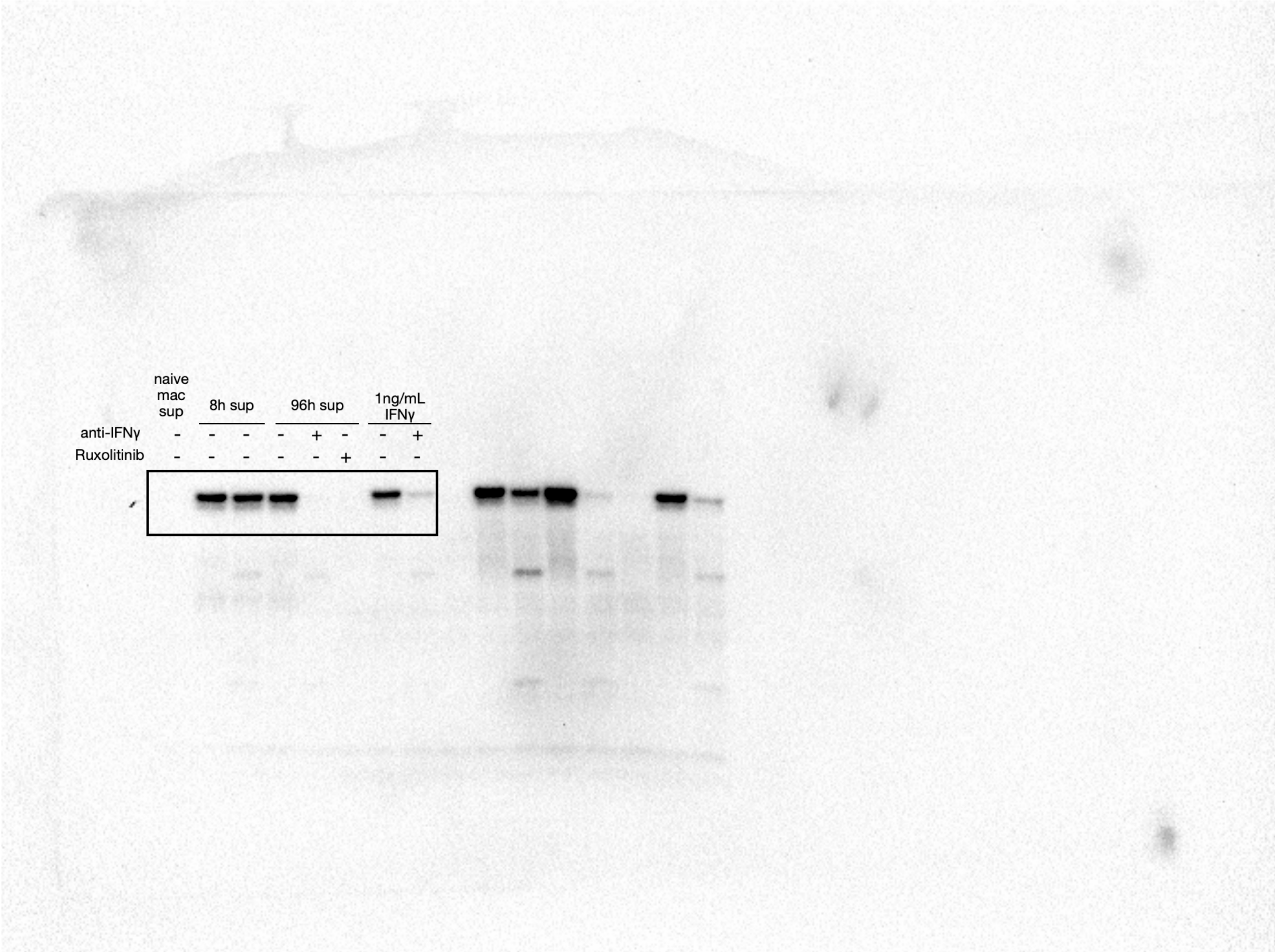

naive mac sup
8h sup
96h sup
1ng/mL IFNγ
anti-IFNγ - - - - + - - +
Ruxolitinib - - - - - + - -

F3D_GAPDH

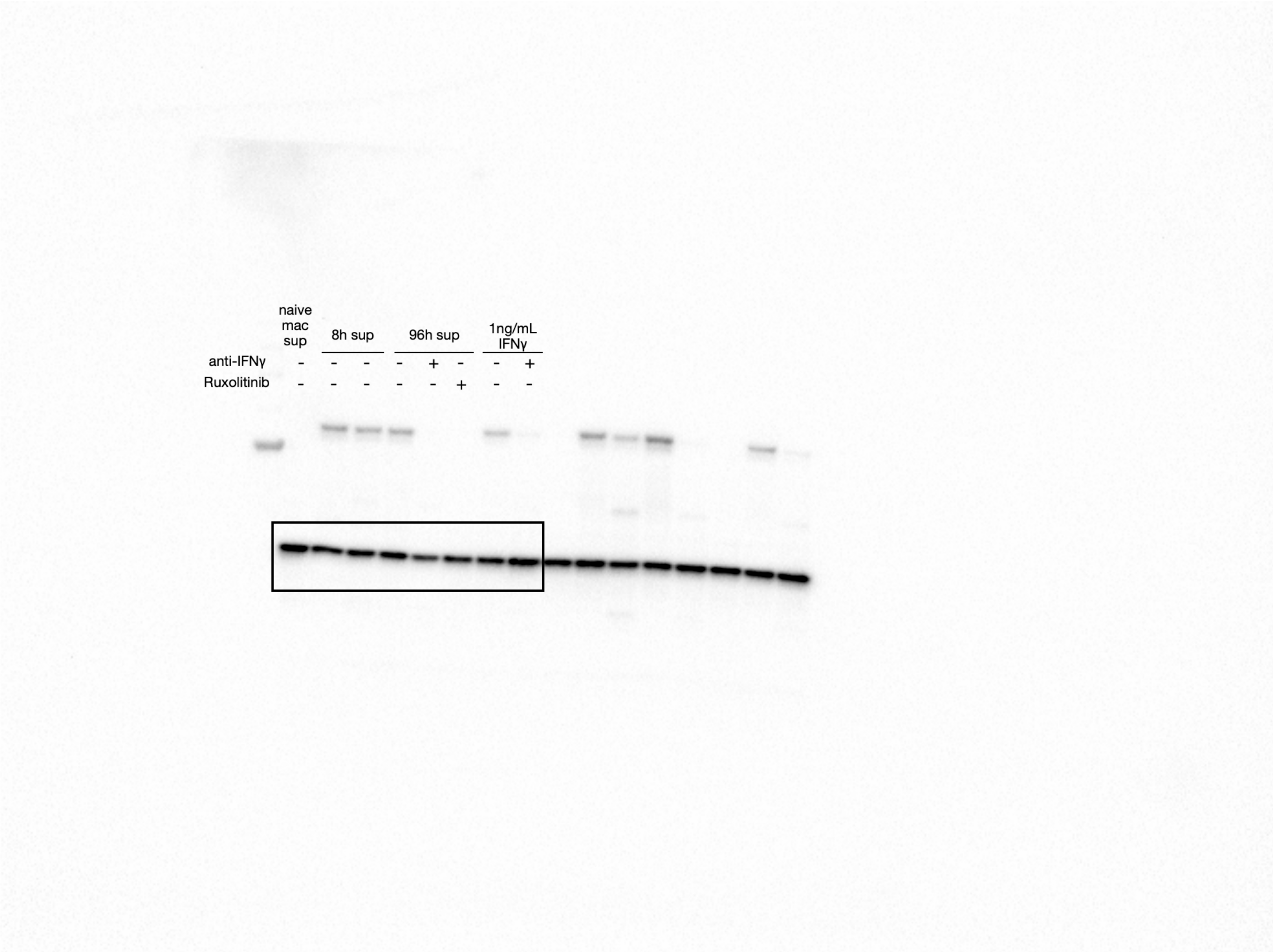
naive mac sup
8h sup
96h sup
1ng/mL IFNγ
anti-IFNγ
- - - - + - - +
Ruxolitinib
- - - - - + - -

F3E_pSTAT1

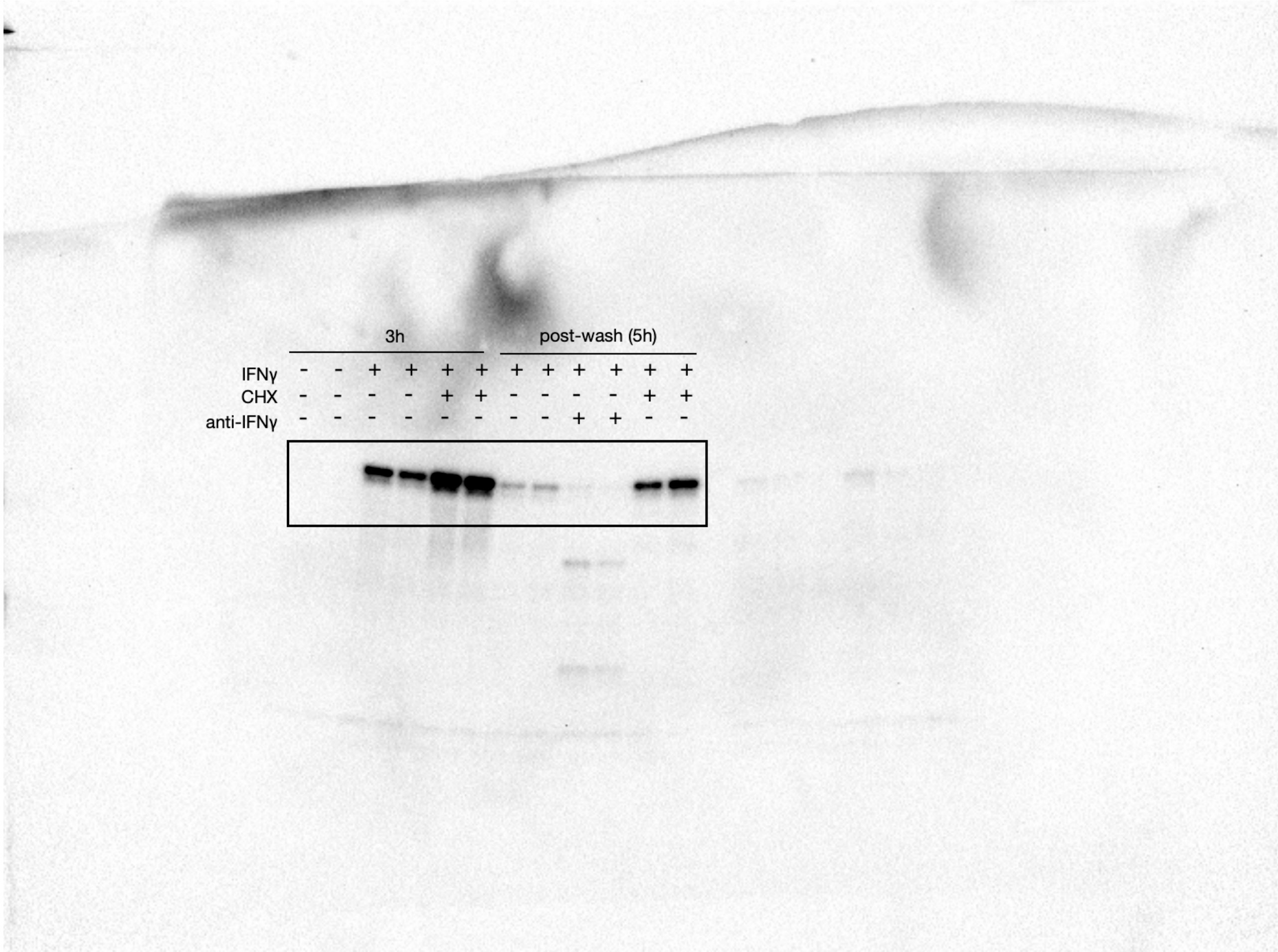

F3E_GAPDH

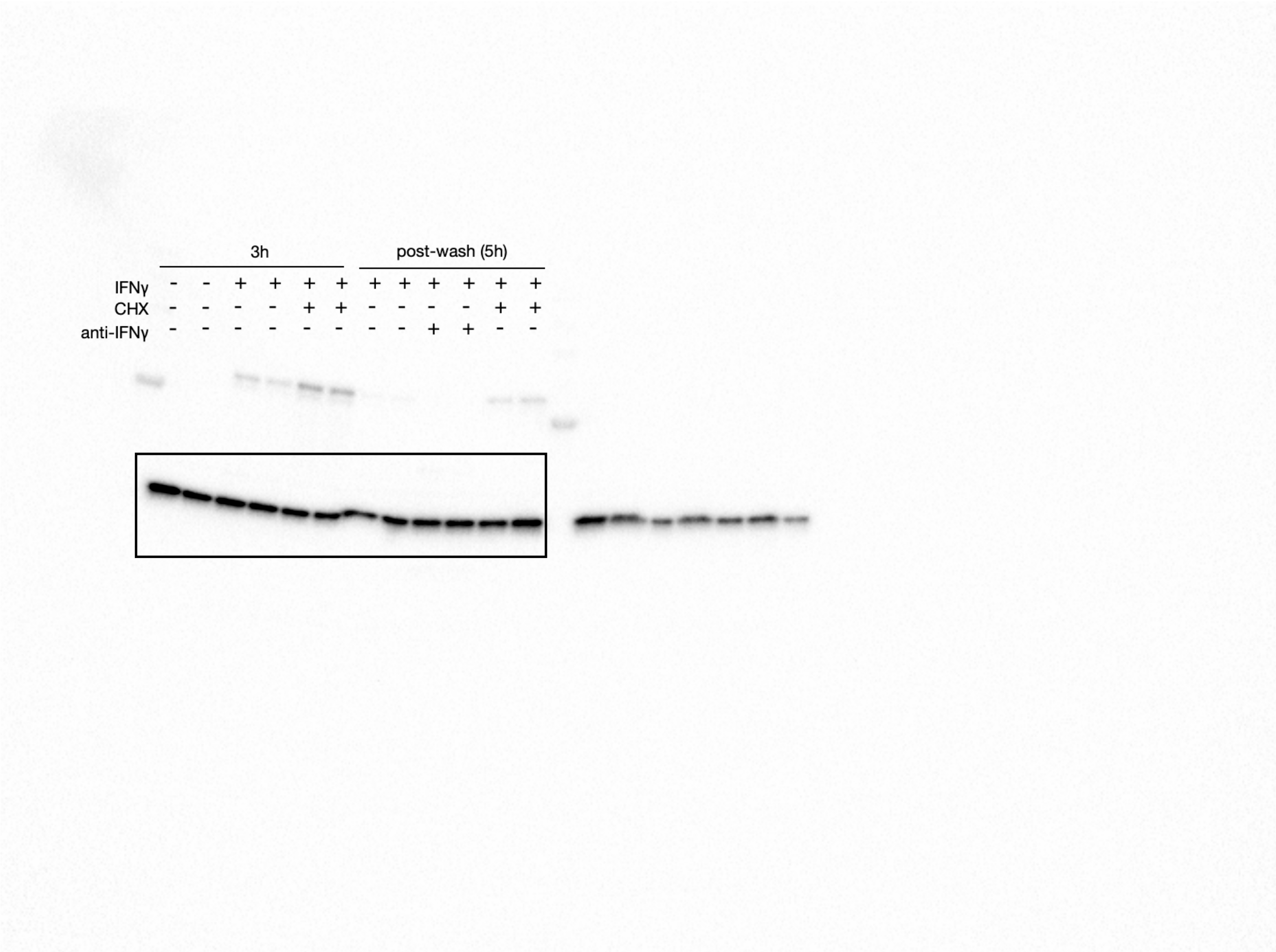

F3G_pSTAT1

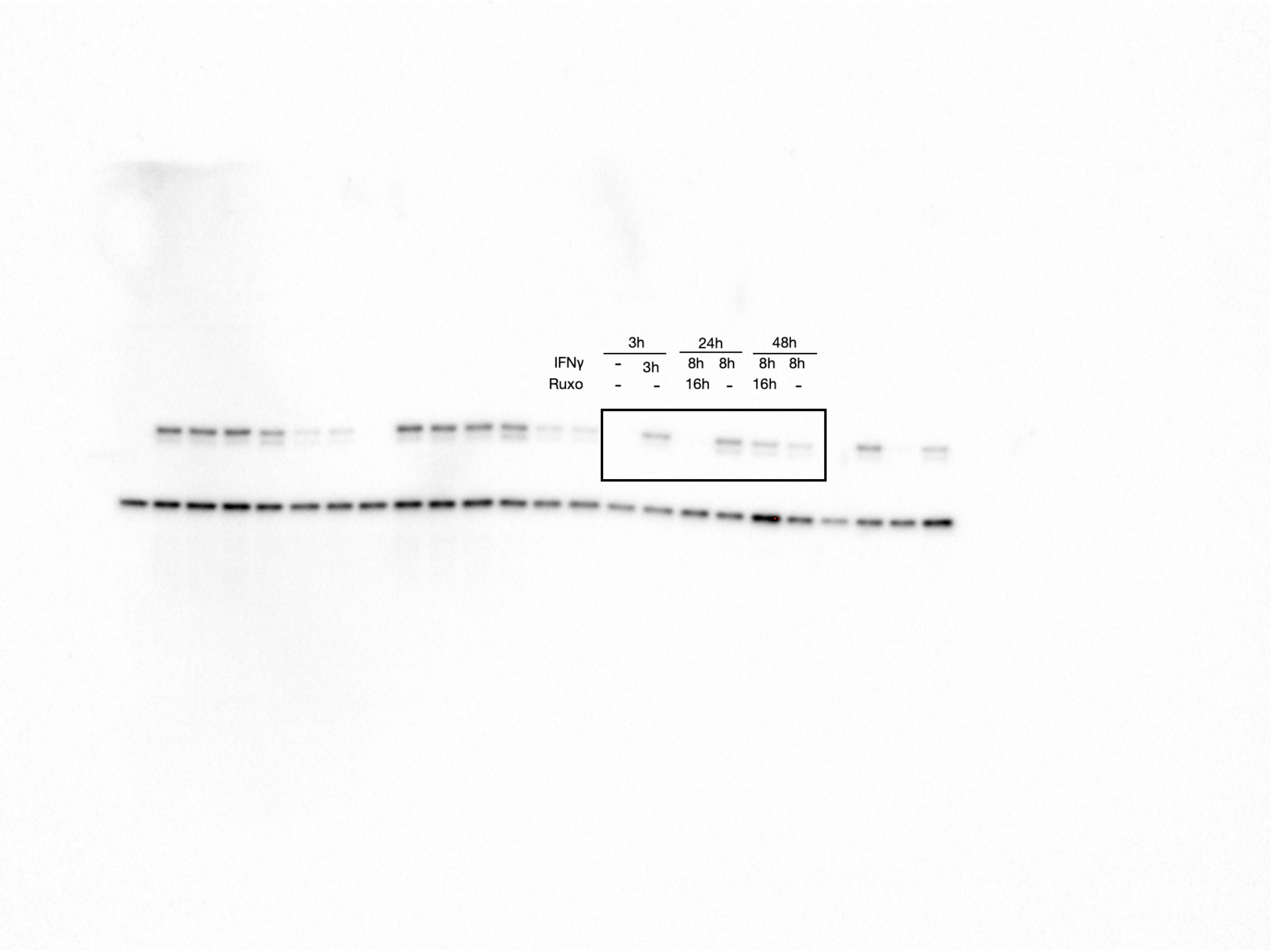

F3G_tubulin

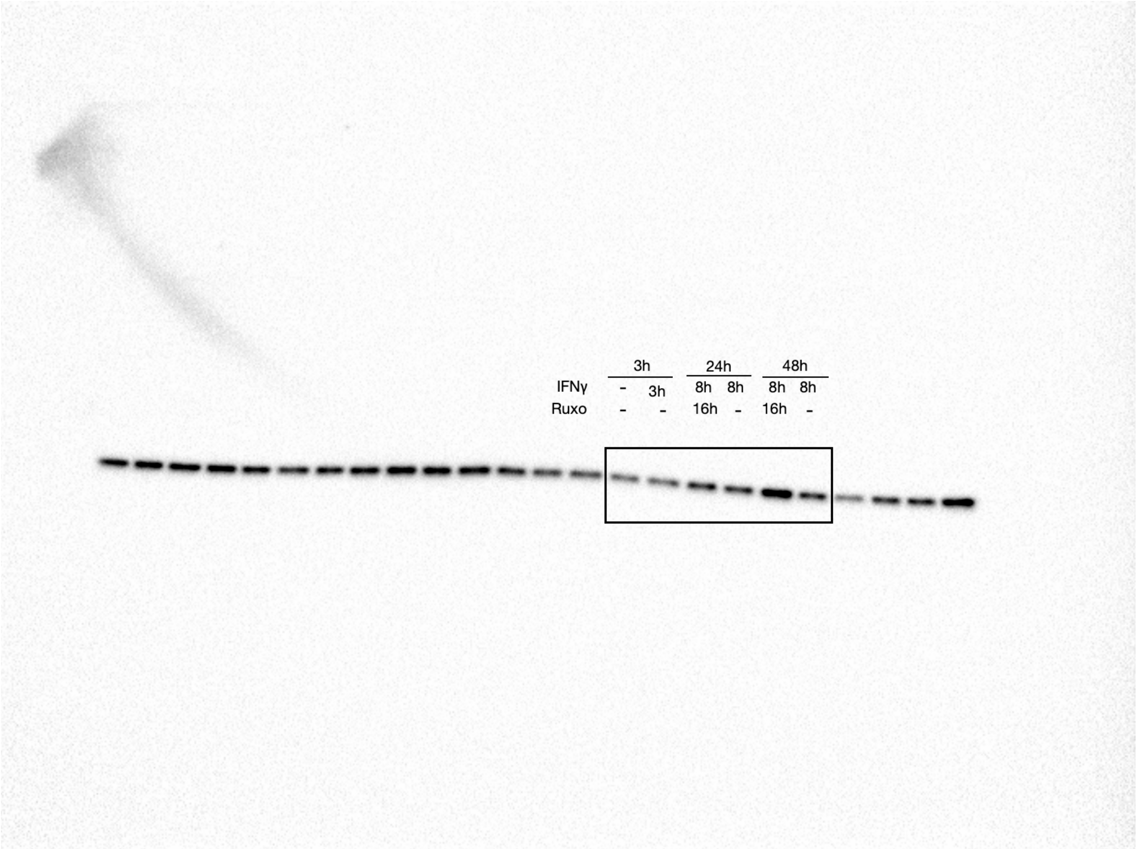

Supplement: SourceData F3 — is the source file for Fig. 3. [file jem_20250976_sourcedataf3.pdf]
